# Prioritizing putative influential genes in cardiovascular disease susceptibility by applying tissue-specific Mendelian randomization

**DOI:** 10.1186/s13073-019-0613-2

**Published:** 2019-01-31

**Authors:** Kurt Taylor, George Davey Smith, Caroline L. Relton, Tom R. Gaunt, Tom G. Richardson

**Affiliations:** 1 MRC Integrative Epidemiology Unit, Bristol Medical School (Population Health Sciences), University of Bristol, Oakfield House, Oakfield Grove, Bristol, BS8 2BN UK; 2 National Institute for Health Research Biomedical Research Centre, Bristol, UK

**Keywords:** Gene expression, DNA methylation, Tissue specificity, Cardiovascular disease, Mendelian randomization, Quantitative trait loci, ALSPAC, ARIES

## Abstract

**Background:**

The extent to which changes in gene expression can influence cardiovascular disease risk across different tissue types has not yet been systematically explored. We have developed an analysis pipeline that integrates tissue-specific gene expression, Mendelian randomization and multiple-trait colocalization to develop functional mechanistic insight into the causal pathway from a genetic variant to a complex trait.

**Methods:**

We undertook an expression quantitative trait loci-wide association study to uncover genetic variants associated with both nearby gene expression and cardiovascular traits. Fine-mapping was performed to prioritize possible causal variants for detected associations. Two-sample Mendelian randomization (MR) was then applied using findings from genome-wide association studies (GWAS) to investigate whether changes in gene expression within certain tissue types may influence cardiovascular trait variation. We subsequently used Bayesian multiple-trait colocalization to further interrogate the findings and also gain insight into whether DNA methylation, as well as gene expression, may play a role in disease susceptibility. Finally, we applied our analysis pipeline genome-wide using summary statistics from large-scale GWAS.

**Results:**

Eight genetic loci were associated with changes in gene expression and measures of cardiovascular function. Our MR analysis provided evidence of tissue-specific effects at multiple loci, of which the effects at the *ADCY3* and *FADS1* loci for body mass index and cholesterol, respectively, were particularly insightful. Multiple-trait colocalization uncovered evidence which suggested that changes in DNA methylation at the promoter region upstream of *FADS1*/*TMEM258* may also affect cardiovascular trait variation along with gene expression. Furthermore, colocalization analyses uncovered evidence of tissue specificity between gene expression in liver tissue and cholesterol levels. Applying our pipeline genome-wide using summary statistics from GWAS uncovered 233 association signals at loci which represent promising candidates for further evaluation.

**Conclusions:**

Disease susceptibility can be influenced by differential changes in tissue-specific gene expression and DNA methylation. The approach undertaken in our study can be used to elucidate mechanisms in disease, as well as helping prioritize putative causal genes at associated loci where multiple nearby genes may be co-regulated. Future studies which continue to uncover quantitative trait loci for molecular traits across various tissue and cell types will further improve our capability to understand and prevent disease.

**Electronic supplementary material:**

The online version of this article (10.1186/s13073-019-0613-2) contains supplementary material, which is available to authorized users.

## Background

Despite recent efforts in research and development, cardiovascular disease still poses one of the greatest threats to public health throughout the world, accounting for more deaths than any other cause [1]. Since their development, genome-wide association studies (GWAS) have identified thousands of different genetic loci associated with complex disease traits [2]. An example of their successful application within cardiovascular research is the identification of numerous genetic variants associated with low-density lipoprotein (LDL) cholesterol levels [3], which is a causal mediator along the coronary heart disease progression pathway [4, 5]. However, the functional and clinical relevance for the vast majority of GWAS results are still unknown, emphasizing the importance of developing our understanding of the causal pathway from single nucleotide polymorphism (SNP) to disease.

A large proportion of associations detected by GWAS are located in non-coding regions of the genome [6], suggesting that the underlying SNPs influence complex traits via changes in gene regulation [7]. Recent efforts have incorporated messenger ribonucleic acid (mRNA) expression data into analyses to determine whether SNPs identified by GWAS influence levels of gene expression (i.e. whether they are expression quantitative trait loci (eQTL)) [8]. Novel methods have integrated eQTL data with summary association statistics from GWAS [9] to identify genes whose nearby (*cis*) regulated expression is associated with traits of interest (widely defined as variants within 1 Mb on either side of a gene’s transcription start site (TSS)) [10]. These are referred to as transcriptome-wide association studies (TWAS). TWAS include distinct methods to predict gene expression using reference panels. In this study, we have performed a similar analysis, except instead of using predicted gene expression, we have identified eQTL from a reference population and evaluated their direct association with complex traits. Therefore, to differentiate between this approach and TWAS, we describe the approach as an eQTL-wide association study (eQTLWAS).

A recent paper has highlighted some limitations that may be encountered by studies integrating transcriptome data to infer causality [11], such as intra-tissue variability and co-regulation amongst proximal genes, making it challenging to disentangle putative causal genes for association signals. This exemplifies the importance of developing methods that investigate tissue specificity and co-regulation of association signals. Therefore, there needs to be further research into the most appropriate manner to harness eQTL data (across multiple tissue and cell types) in order to improve the biological interpretation of GWAS findings.

**Fig. 1:**
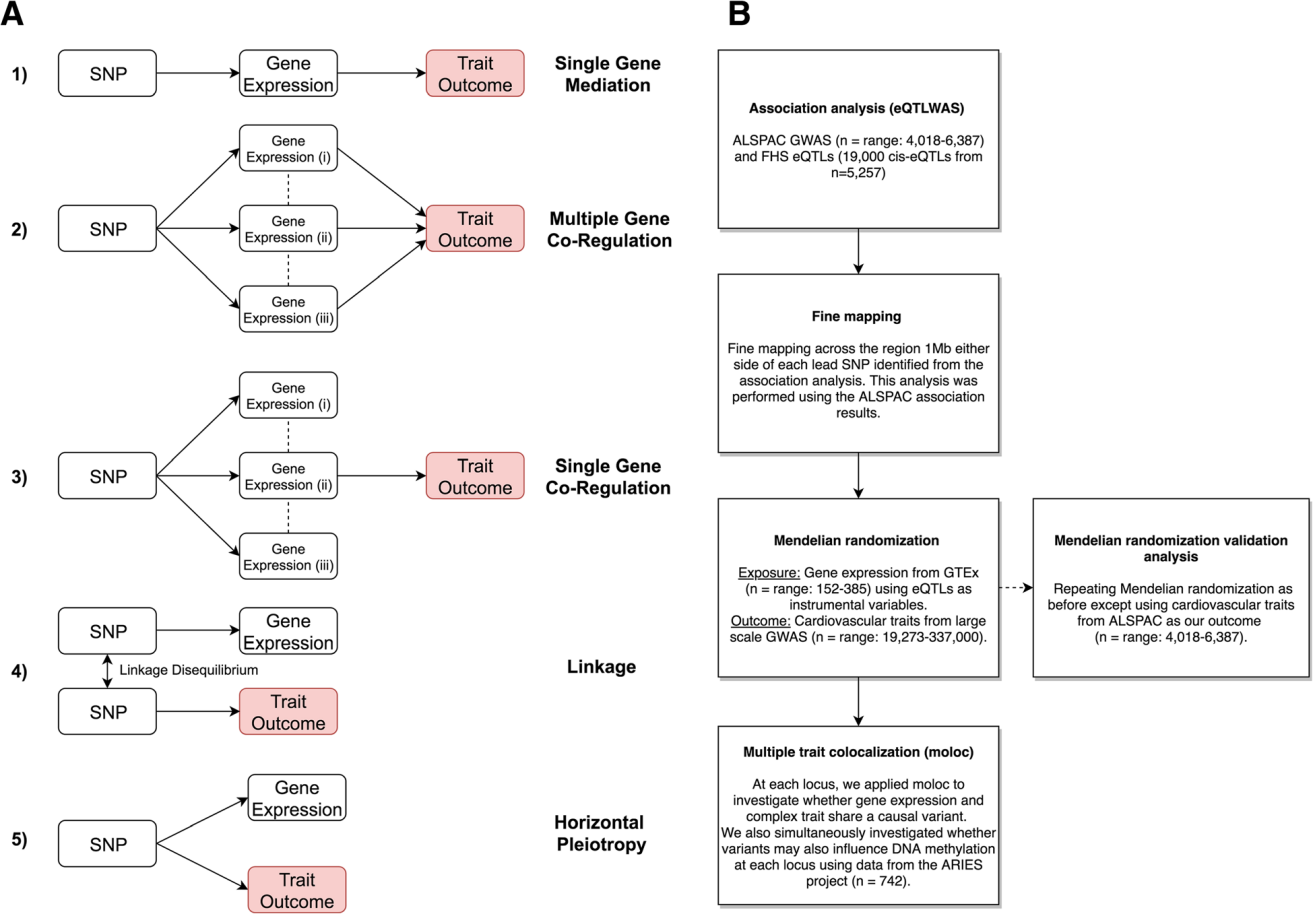
Analysis pipeline and explanations for observed associations between single nucleotide polymorphisms (SNPs) and traits. **a** Five potential scenarios that could explain findings from the expression quantitative trait loci-wide association study (eQTLWAS): 1) The genetic variant influences the trait, mediated by the expression of a single gene at a locus. 2) The genetic variant influences the trait via multiple genes which are co-regulated with one another. 3) The genetic variant influences the trait via a single gene which is co-regulated with other non-causal genes. 4) The genetic variant that influences the trait is in linkage disequilibrium with another variant which is responsible for the changes in gene expression levels. 5) The genetic variant influences both gene expression and the trait outcome by two independent biological pathways (horizontal pleiotropy). **b** Flow diagram illustrating the analysis pipeline used to interrogate the causal pathway from SNP to trait. eQTLWAS was performed to uncover genetic variants associated with nearby gene expression and complex trait. Fine-mapping was implemented to identify potential causal variants. Mendelian randomization (MR) analyses were performed to interrogate scenarios 1, 2 and 3. Multiple-trait colocalization explored shared causal variants between traits (scenario 4). We were unable to investigate horizontal pleiotropy due to an insufficient number of instruments (scenario 5)

In this study, we have developed an analysis pipeline to investigate trait-associated eQTL and postulate five potential scenarios that can help explain them (Fig. 1). Firstly, we identify associations between lead eQTL based on a reference population (such as the Framingham Heart Study (*n* = 5257) [8] used in this study) and complex traits. Regions where associations are detected in this analysis are fine mapped to prioritize causal variants responsible for effects. We then investigate the relationship between gene expression and complex traits at the loci of interest by applying the principles of Mendelian randomization (MR), a method which uses genetic variants associated with an exposure as instrumental variables to infer causality amongst correlated traits [12, 13]. A recent development in this paradigm is two-sample MR, by which effect estimates on exposures and outcomes are derived from two independent datasets, allowing researchers to exploit findings from large GWAS consortia [14]. Applying this approach can therefore be used to help infer whether changes in gene expression (our exposure) may influence a complex trait identified by GWAS (our outcome) (Additional file 1). Furthermore, as tissue specificity is fundamental to understanding causal mechanisms involving gene expression, we have used data from the Genotype Tissue Expression Project (GTEx) [15] in a number of tissues that could be important in cardiovascular disease susceptibility (Additional file 2: Table S1) to try and disentangle co-regulation amongst proximal genes (i.e. differentiating between scenarios 1, 2 and 3 (Fig. 1a)). We refer to this approach as tissue-specific MR, which should prove increasingly valuable in investigating both the determinants and consequences of changes in tissue-specific gene expression as sample sizes increase [12].

We subsequently apply colocalization analyses [16] at each locus of interest to evaluate whether gene expression and complex trait share the same causal variant. This is opposed to the alternative explanation, which is that association signals may be a product of linkage disequilibrium (LD) between two separate causal variants (Fig. 1a: scenario 4). This analysis can also complement the findings from the MR analysis, particularly given that the majority of genes can only be instrumented with a single eQTL using GTEx data. In addition, there has been a recent interest in the impact that DNA methylation may have on cardiovascular disease risk via modifications in gene expression [17]. Therefore, we apply multiple-trait colocalization (moloc) [16] at each locus to simultaneously investigate whether the same underlying genetic variant is driving the observed effect on all three traits of interest (i.e. the cardiovascular trait, gene expression and DNA methylation).

Uncovering evidence suggesting that DNA methylation and gene expression may be working in harmony to influence complex traits can improve the reliability of causal inference in this field, as it suggests there may be underlying mechanisms which are consistent with causality (e.g. DNA methylation acting as a transcriptional repressor). However, a major challenge in this paradigm is the lack of accessible tissue-specific DNA methylation/methylation QTL (mQTL) data akin to GTEx for gene expression. Previous studies have investigated the potential mediatory role of DNA methylation between a genetic variant and gene expression using eQTL and mQTL data derived from the blood which may act as a proxy for other tissue types [18–20]. Moreover, other studies have demonstrated a surprisingly high rate of replication between mQTL derived from the blood and more relevant tissue types for a complex trait of interest [21]. We have therefore undertaken moloc analyses using eQTL derived from both blood and cardiovascular-specific tissue types. Finally, it is also important to note that, along with other approaches which apply causal methods to molecular data, we are currently unable to robustly differentiate mediation from horizontal pleiotropy (Fig. 1a: scenario 5) [12, 22]. However, within this pipeline, we will be able to accommodate additional eQTL as instrumental variables derived from future larger studies in order to address this.

In this study, we demonstrate the value of our pipeline by undertaking an applied investigation attempting to link genetic variants to cardiovascular risk factors via changes in gene regulation. We have used the Avon Longitudinal Study of Parents and Children (ALSPAC) as our discovery cohort which has early life measures of cardiovascular function that were analysed as complex traits. Associations detected between eQTL and cardiovascular traits early in the life course can be extremely valuable for disease prevention and healthcare. We used ~ 19,000 *cis*-eQTLs observed in adults at risk of cardiac events from the Framingham Heart Study [8] for our eQTLWAS to ascertain whether they influence these cardiovascular traits in young individuals (age ≤ 10 in ALSPAC). We have further evaluated the results using our analysis pipeline (Fig. 1b) by harnessing summary statistics from large-scale GWAS to demonstrate the value of our approach and validate findings in independent samples. Finally, we apply our pipeline in a genome-wide manner using the findings from large-scale GWAS, which may prove useful in prioritizing putative causal genes for further evaluations by future studies.

## Methods

### The Avon Longitudinal Study of Parents and Children

Detailed information about the methods and procedures of ALSPAC is available elsewhere [23–25]. In brief, ALSPAC is a prospective birth cohort study which was devised to investigate the environmental and genetic factors of health and development. In total, 14,541 pregnant women with an expected delivery date of April 1991 and December 1992, residing in the former region of Avon, UK, were eligible to take part. Participants attended regular clinics where detailed information and bio-samples were obtained. The study website contains details of all the data that is available through a fully searchable data dictionary [26]. All procedures were ethically approved by the ALSPAC Ethics and Law Committee and the Local Research Ethics Committees. Written informed consent was obtained from all participants.

#### Genetic data

All children were genotyped using the Illumina HumanHap550 quad genome-wide SNP genotyping platform. Samples were removed if individuals were related or of non-European genetic ancestry. Imputation was performed using Impute V2.2.2 against a reference panel from 1000 Genomes [27] phase 1 version 3 [28]. After imputation, we filtered out variants and kept those with an imputation quality score of ≥ 0.8 and minor allele frequency (MAF) > 0.01.

#### Phenotypes

The methods and procedures to acquire data for the 14 phenotypes analysed in this study are as follows. All measurements were obtained at the ALSPAC clinic. Height and weight were measured at age 7 (mean age 7.5, range 7.1–8.8). Height was measured to the nearest 0.1 cm with a *Harpenden* Stadiometer (*Holtain Crosswell*), and weight was measured to the nearest 0.1 kg on *Tanita* electronic scales. Body mass index (BMI) was calculated as (weight [kg]/(height [m]^2^). Non-fasting blood samples were taken at age 10 (mean age 9.9, range 8.9–11.5). The methods on the assays performed on these samples which included total cholesterol, high-density lipoprotein cholesterol, LDL cholesterol (calculated using the Friedewald equation [29]), very low-density lipoprotein (VLDL) cholesterol, triglycerides, apolipoprotein A1 (ApoA1), apolipoprotein B (ApoB), fasting glucose, fasting insulin, adiponectin, leptin, C-reactive protein (CRP) and interleukin 6 (IL-6) have been described previously [30].

### The Framingham Heart Study

We identified over 19,000 pruned lead *cis*-eQTLs from Joehanes et al. [8] who provide in-depth details of the Framingham Heart Study and their analysis plan in their paper. Trans-eQTLs were not considered for our analysis to reduce the likelihood of horizontal pleiotropy influencing our findings and also to reduce the burden of multiple testing [31]. This eQTL data was chosen for the initial analysis in ALSPAC due to the larger sample size of transcriptome data from the Framingham Heart Study (*n* = 5257) using whole blood in comparison to GTEx sample sizes for other tissue types. This allowed us to maximize statistical power to detect association signals which we were then subsequently able to evaluate in detail using data from other tissue types.

### The Genotype Tissue Expression Project

GTEx is a unique open-access online resource with gene expression data for 449 human donors (83.7% European American and 15.1% African American) across 44 tissues. Sample sizes vary between tissues, thus affecting statistical power to identify eQTL. In-depth information on the materials and methods for GTEx is available in the latest publication [15]. In short, RNA sequencing samples were sequenced to a median depth of 78 million reads. This is suggested to be a credible depth to quantify accurately genes that may have low expression levels [32]. DNA was genotyped at 2.2 million sites and imputed to 12.5 million sites. We used GTEx eQTL data (v6p) in the Mendelian randomization and multiple-trait colocalization analyses (Fig. 1b). The mean donor age for all tissues included in this analysis resided between 50 and 55 years (range 20–79).

### Statistical analysis

Data from ALSPAC were initially cleaned using STATA [version 15], and outliers defined as ± 4 standard deviations from the mean were removed. We plotted histograms to check the data for normality and where necessary applied log transformation. Using PLINK [version 1.9] [33, 34], we undertook an age- and sex-adjusted eQTLWAS to evaluate the association between *cis*-eQTLs known to influence gene expression and cardiovascular traits. We applied a Bonferroni correction to account for multiple testing which equated to 0.05/the total number of tests undertaken. Using a script derived from the qqman R package [35], the results were plotted using a Manhattan plot. We undertook fine-mapping across the region 1 Mb either side of each lead SNP identified from our eQTLWAS using FINEMAP software [36]. As FINEMAP requires LD statistics between variants in a region, we performed this analysis in the ALSPAC dataset where we had individual-level data to compute LD. This was opposed to using the summary-level data from the large-scale GWAS which requires a reference population such as the 1000 Genomes Project as this has been reported to influence false-positive rates [37]. In all FINEMAP analyses, we used the default setting which outputs a maximum of five putative causal variants.

#### Tissue-specific Mendelian randomization analysis

To investigate potential causal genes at association signals detected in our eQTLWAS, we applied the principles of MR using the Wald ratio method [38] (Additional file 1: Fig. S1) to assess whether changes in tissue-specific gene expression (eQTLs as instrumental variables) may be responsible for effects on associated traits. Furthermore, it may help discern whether multiple proximal genes at a region are contributing to trait variation or whether they are co-regulated with the causal gene(s), i.e. scenario 3. Firstly, for each lead eQTL from the eQTLWAS, we used tissue-specific data from GTEx to discern whether they were *cis*-eQTL for genes in tissue types which may play a role in the pathology of cardiovascular disease (*P* < 1 × 10^−4^). The tissue types evaluated were as follows: adipose–subcutaneous; adipose–visceral (omentum); liver, pancreas, artery–coronary; artery–aorta; heart–atrial appendage; and heart–left ventricle. In addition to this, we ran an additional analysis for the association with BMI but investigating the effects in the following brain tissues: pituitary, anterior cingulate cortex (BA24) and frontal cortex (BA9).

For this analysis, we used data from large-scale GWAS; a full list of these with details can be found in Additional file 2: Table S2) [39–41]. These analyses were undertaken using the MR-Base platform [42]. The only trait we were unable to assess in this analysis was IL-6, due to the lack of GWAS summary statistics for this trait. We applied a multiple testing threshold to the MR results based on the number of tests undertaken. We plotted the results from the validation analysis using volcano plots from the ggplot2 package in R [43]. We also applied the Steiger directionality test [44] to discern whether our exposure (i.e. gene expression) was influencing our outcome (i.e. our complex trait) as opposed to the opposite direction of effect. The null hypothesis for this test is that the *r*^2^ for the SNP-exposure and SNP-outcome effects are the same. The alternative hypothesis is that they are different more than we would expect by chance.

We then undertook a validation analysis by repeating MR analyses using ALSPAC data. As cardiovascular trait data is therefore obtained at an earlier stage in the life course compared to the tissue-specific expression data, any associations detected in the validation analysis suggest genetic liability to cardiovascular risk via changes in gene expression.

#### Multiple-trait colocalization

Blood samples were obtained from 1018 ALSPAC mothers as part of the Accessible Resource for Integrated Epigenomics Studies (ARIES) [45] from the ‘Focus on Mothers 1’ time point (mean age = 47.5). Epigenome-wide DNA methylation was derived from these samples using the Illumina HumanMethylation450 (450K) BeadChip array (*n* = 742 after quality control). From this data, we obtained effect estimates for all genetic variants within a 1-Mb distance of lead eQTL from the eQTLWAS and proximal CpG sites (again defined as < 1 Mb). We then used the moloc [16] method to investigate two questions:

1. Is the same underlying genetic variant influencing changes in both proximal gene expression and cardiovascular trait (i.e. investigating scenario 4 (Fig. 1a))?
2. Does the genetic variant responsible for these changes also appear to influence proximal DNA methylation levels, suggesting that changes in this molecular trait may also play a role along the causal pathway to disease?

As such, at each locus, we applied moloc using genetic effects on 2 different molecular phenotypes (gene expression and DNA methylation (referred to as eQTL and mQTL respectively)) along with the associated cardiovascular trait from our GWAS summary statistics. Since we evaluated three traits in this analysis (i.e. gene expression, DNA methylation and cardiovascular trait), moloc computed 15 possible configurations of how genetic variation may influence these traits (Additional file 2: Table S3). Detailed information on how these are calculated can be found in the original moloc paper [16]. For each independent trait-associated locus, we extracted effect estimates for all variants within 1 Mb distance of the lead eQTLWAS hit, for all molecular phenotypes and relevant cardiovascular GWAS traits. We subsequently applied moloc in a gene-centric manner, by mapping CpG sites to genes based on the 1-Mb regions either side of our eQTLWAS hit. This approach was applied to all gene-CpG combinations, within each region of interest. We ran this analysis twice, once using expression data from whole blood and again using expression data from a tissue type which was associated with the corresponding trait in the tissue-specific MR analysis (Additional file 2: Table S4).

Only the regions with at least 50 SNPs (MAF ≥ 5%) in common between all three datasets (i.e. gene expression, DNA methylation and cardiovascular trait) were assessed by moloc based on the recommendations by the authors. We computed summed posterior probability of associations (PPAs) for all scenarios where GWAS trait and gene expression colocalized. When summed PPAs were ≥ 0.8, we reported the findings as evidence that genetic variation was influencing cardiovascular traits via changes in gene expression. Furthermore, when summed PPAs relating to DNA methylation were ≥ 0.8, there was evidence that DNA methylation may also reside on the causal pathway to complex trait variation via changes in gene expression. In all analyses, we used prior probabilities of 1e−04, 1e−06 and 1e−07 as recommended by the developers of moloc based on their simulations [16].

#### Applying the analytical pipeline genome-wide using findings from large-scale consortia

Finally, we demonstrate how our approach can be applied genome-wide using summary level statistics available from large-scale GWAS consortia. Lead eQTLs were identified across nine tissue types from the GTEx consortium (the eight described above as well as whole blood), and their association with complex traits was evaluated using findings from the GWAS described in Additional file 2: Table S2. When an eQTL could not be located using these summary statistics, we attempted to locate a proxy SNP (i.e. *r*^2^ ≥ 0.8) based on a reference panel of European individuals from the 1000 Genomes Phase 3 Project [27].

Associations between eQTL and complex traits which survived multiple testing were analysed using MR. Furthermore, moloc was applied as before using data from the ARIES project. Fine-mapping was not undertaken in this analysis as we did not have individual-level data from these GWAS, and using a reference panel in these situations has been reported to increase the likelihood of spurious findings [37].

## Results

### Identifying putative causal genes for measures of cardiovascular function

**Fig. 2:**
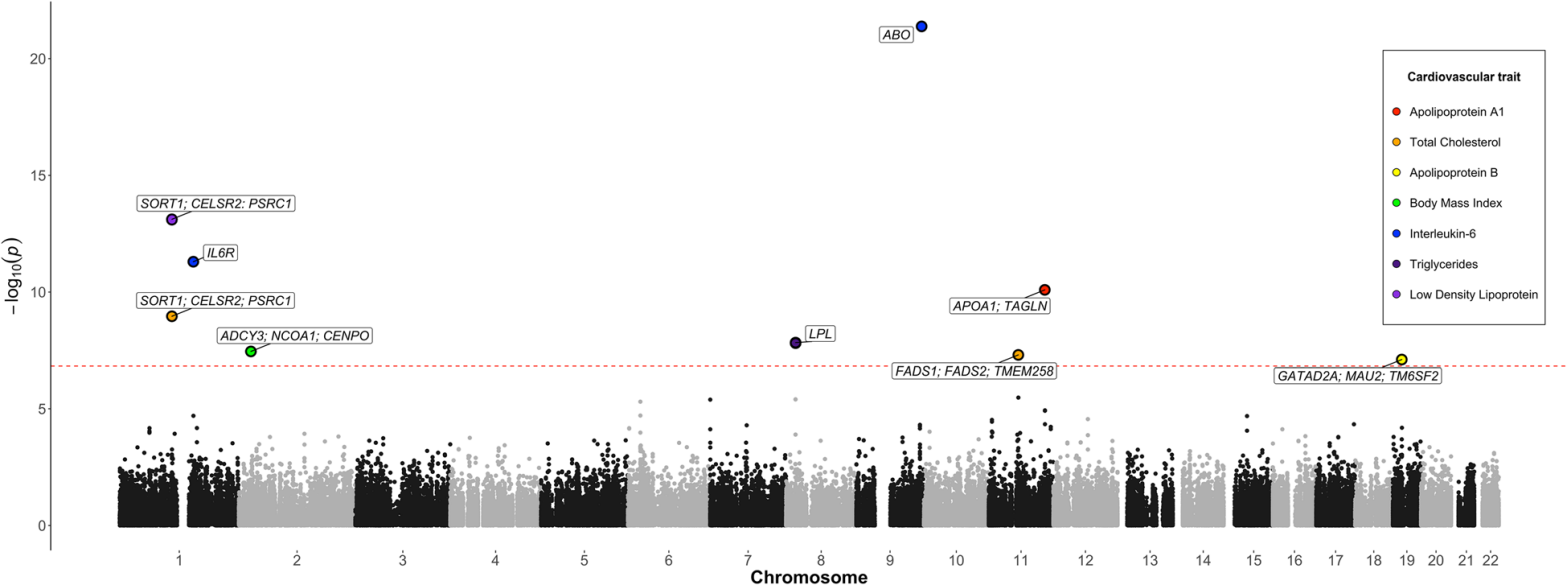
Manhattan plot illustrating observed associations between expression quantitative trait loci (eQTLs) and cardiovascular traits in the ALSPAC cohort. Analyzed SNPs are plotted on the *x*-axis ordered by chromosomal position against −log10 *P* values which are plotted on the *y*-axis. SNPs that survived the multiple testing threshold (1.8 × 10^−7^—represented by the red horizontal line) are coloured according to their associated trait and annotated with potential causal gene symbols

We carried out 273,742 tests to evaluate the association between previously identified *cis*-eQTLs [8] with 14 cardiovascular traits in turn within ALSPAC (19,553 *cis*-eQTLs × 14 traits). After multiple testing corrections, we identified 11 association signals across 8 unique genetic loci which provided strong evidence of association (*P* < 1.8 × 10^−7^ [Bonferroni-corrected threshold, *P* < 0.05/273,742]). These results can be found in Table 1 and are illustrated in Fig. 2. The region near *SORT1* was associated with total cholesterol, LDL cholesterol and ApoB. Additionally, the *LPL* region was associated with both triglycerides and VLDL cholesterol.

**Table 1:** Results of the expression quantitative trait loci-wide association study (eQTLWAS) between genetic variants influencing gene expression and cardiovascular traits in ALSPAC

Tag SNP	Gene(s)	Trait	Sample size	Beta	SE	*P* value
rs646776	*SORT1*; *CELSR2*; *PSRC1*	Total cholesterol	4543	− 0.099	0.016	1.10 × 10^−9^
rs646776	*SORT1*; *CELSR2*; *PSRC1*	LDL cholesterol	4543	− 0.110	0.015	7.74 × 10^−14^
rs646776	*SORT1*; *CELSR2*; *PSRC1*	ApoB	4546	− 2.695	0.328	2.66 × 10^−16^
rs12129500	*IL6R*	IL-6	4503	− 0.126	0.018	4.96 × 10^−12^
rs11693654	*ADCY3*; *NCOA1*; *CENPO*	BMI	6387	0.200	0.036	3.57 × 10^−8^
rs80026582	*LPL*	Triglycerides	4334	− 0.101	0.018	1.49 × 10^−8^
rs80026582	*LPL*	VLDL cholesterol	4334	− 0.100	0.018	1.57 × 10^−8^
rs600038	*ABO*	IL-6	4496	− 0.207	0.021	4.12 × 10^22^
rs174538	*FADS1*; *FADS2*; *TMEM258*	Total cholesterol	4539	− 0.080	0.015	5.03 × 10^−8^
rs2727784	*APOA1*; *TAGLN*	ApoA1	4018	3.047	0.468	8.05 × 10^−11^
rs10419998	*GATAD2A*; *MAU2*; *TM6SF2*	ApoB	4404	− 2.024	0.376	7.96 × 10^−8^

Abbreviations for the column headings from left to right: single nucleotide polymorphism, gene or gene cluster associated with SNP, associated trait, sample size for this effect, observed effect size, standard error of the effect size, *P* value for the observed effect

We undertook fine-mapping 1 Mb either side of the lead SNP at each locus identified in our initial analysis to investigate which SNP(s) may be driving the observed effects of complex traits. PPAs from FINEMAP [36] suggested that there was most likely only a single variant influencing trait variation for 7 of the 11 total loci. For the other 4 loci, FINEMAP suggested there may be multiple variants influencing traits (Additional file 2: Table S5).

### Disentangling causal mechanisms using tissue-specific Mendelian randomization

We undertook 54 MR analyses across all tissues and complex traits from large-scale GWAS and identified 34 associations between tissue-specific gene expression and cardiovascular traits (*P* < 9.3 × 10^−4^ (i.e. 0.05/54 tests) (Additional file 2: Tables S6-S16). In the separate validation analysis in ALSPAC, we observed consistent directions of effect for 30 of the associations. The potential value of this approach in terms of disentangling causal genes (i.e. scenarios 2 and 3 (Fig. 1a)) was exemplified at the BMI-associated region on chromosome 2. Of the 3 *cis*- and potentially causal genes for this signal, only *ADCY3* provided strong evidence of being the putative causal gene in two types of adipose tissue (adipose subcutaneous (*P* = 6.8 × 10^−40^) and adipose visceral (*P* = 3.1 × 10^−48^)) (Additional file 1: Fig. S2). This suggests that changes in *ADCY3* expression in adipose tissue could influence BMI levels. In contrast, there was a lack of evidence that changes in *NCOA1* expression in the analysed tissue types influence BMI. We were unable to undertake MR of *CENPO* expression in this analysis as were unable to harmonize the effect estimates between exposure and outcome. As an additional analysis, we repeated the MR on BMI using eQTL effect estimates derived from *ADCY3* expression in brain tissue (pituitary), although there was limited evidence of association (beta (SE), 0.008 (0.006); *P*, 0.177).

Additional file 1: Fig. S2 also illustrates the results observed at the cholesterol associated region on chromosome 11. There was evidence that *FADS1* expression was associated with total cholesterol in three different tissues (adipose subcutaneous (*P* = 2.2 × 10^−40^), heart left ventricle (*P* = 1.0 × 10^−35^) and pancreas (P = 2.2 × 10^−40^)). Interestingly, the strength of evidence was comparable between subcutaneous adipose and pancreas tissues despite the differences in GTEx sample sizes (pancreas 220 and adipose subcutaneous 385) (Additional file 1: Fig. S3). *TMEM258* expression provided strong evidence of association in one tissue type (adipose subcutaneous (*P* = 7.2 × 10^−34^)), whereas association between *FADS2* expression and total cholesterol was observed in multiple tissue types (adipose—subcutaneous (*P* = 5.1 × 10^−11^), adipose—visceral (*P* = 4.2 × 10^−20^), artery—aorta (*P* = 5.8 × 10^−10^), heart—atrial appendage (*P* = 6.3 × 10^−5^) and pancreas (*P* = 6.3 × 10^−5^)). The most parsimonious explanation may be that multiple genes at this locus influence cholesterol levels; however, further analyses are required to robustly differentiate between scenarios 2 and 3 here (Fig. 1a).

At other loci evaluated (Additional file 1: Figures S4-S10), *LPL* showed evidence of association with triglycerides in a single tissue (adipose subcutaneous (*P* = 9.6 × 10^−168^)) implying that this effect may be more tissue-specific compared to those observed at other loci in this study (Additional file 1: Figs. S9 and S10, Additional file 2: Tables S15 and S16). On chromosome 1, there was strong evidence that gene expression in the liver influences total cholesterol (Additional file 1: Fig. S7) and LDL (Additional file 1: Fig. S8) (*P* < 3.22 × 10^−120^). However, this was observed for all three genes in the region (*SORT1*, *CELSR2* and *PSRC1*). In these analyses alone, we were unable to determine whether a particular gene is driving this observed effect, with the other proximal genes being co-regulated, or whether there are multiple causal genes for these traits (i.e. scenario 2 (Fig. 1a)). However, evidence from the literature implicates *SORT1* as the most likely causal gene for this association signal [11, 46]*.* Our MR results from ALSPAC provided evidence between *ABO* expression and IL-6 in four different tissues (Additional file 2: Table S13). Although, caution is required when interpreting this signal based on previous evidence across a diverse range of traits [47]. Finally, to test the direction of effect at each locus (i.e. are changes in gene expression causing changes in trait or vice versa), we applied the causal direction test [44]. In all scenarios, the test provided evidence that gene expression influences traits at these loci rather than the opposite direction of effect (Additional file 2: Tables S6-S16).

### Using multiple-trait colocalization to uncover evidence of shared causal variants between cardiovascular and intermediate traits

We identified evidence of colocalization (PPA = ≥ 0.8) for seven unique genes across five loci across various tissue types (Additional file 2: Tables S17-S21). Building upon the results from the tissue-specific MR analysis, we found strong evidence that *ADCY3* is the functional gene for the BMI-associated signal on chromosome 2 (maximum PPA of 0.99 between gene expression and BMI [hypothesis 11: see Additional file 2: Table S3 for explanation]). We identified evidence of colocalization between BMI and *ADCY3* expression in both whole blood and subcutaneous adipose tissue. There was also evidence that distributions between DNA methylation at cg04553793 (at the promoter region of *ADCY3)* colocalized with BMI and *ADCY3* expression in whole blood (PPA = 0.88 [hypothesis 14]). However, the lead mQTL for this observed effect (rs13401333) was not correlated with the lead eQTL and GWAS hit (rs6745073, *r*^2^ = 0.02), which suggests that in-depth analysis with multiple tissue types is necessary to confirm whether DNA methylation influences disease susceptibility at this locus.

**Fig. 3:**
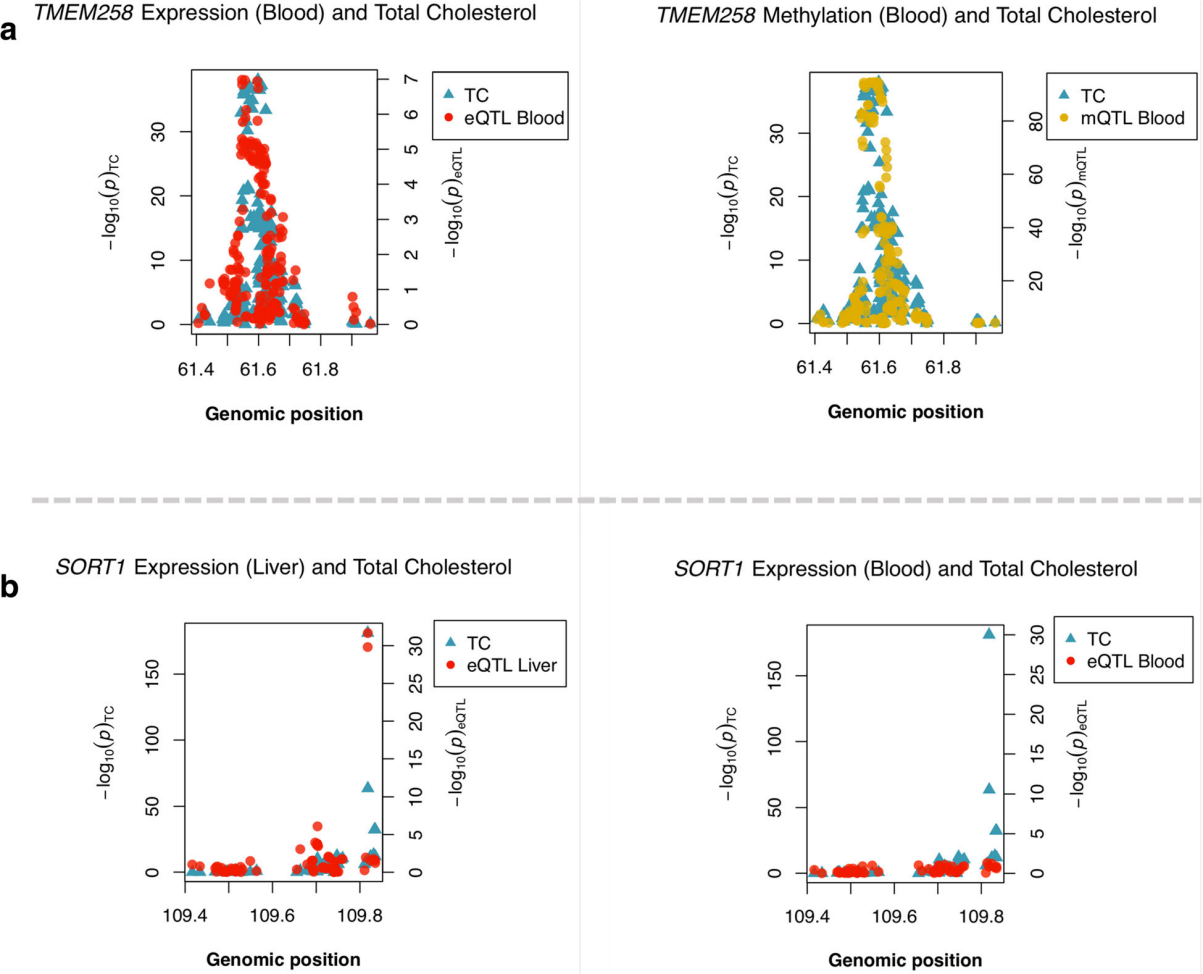
Multiple-trait colocalization analyses between cardiovascular traits and molecular phenotypes. **a** Evidence of colocalization between *TMEM258* expression and total cholesterol (left) as well as DNA methylation at cg19610905 and total cholesterol (right) using data derived from whole blood. **b** Evidence of colocalization between *SORT1* expression using data derived from the liver and total cholesterol (left). However, this evidence diminished when undertaking the same analysis for *SORT1* expression data derived from whole blood (right). Abbreviations: TC, total cholesterol; eQTL, expression quantitative trait loci; mQTL, methylation quantitative trait loci

There was also evidence that changes in DNA methylation at a CpG site in the promoter region for *FADS1* (cg19610905) colocalized with total cholesterol variation. There was evidence of colocalization for all three traits using gene expression for *TMEM258* (PPA = 0.85) (Fig. 3a), where the lead GWAS variant (rs174568) and mQTL were in perfect LD (rs1535, *r*^2 2^ = 1). This effect was only observed in whole blood. Evidence of colocalization between all three traits using *FADS1* expression narrowly missed the cut-off (PPA = 0.77). Finally, we found limited evidence that changes in DNA methylation at this CpG site colocalized with *FADS2* expression, although as with the previously evaluated locus, this was not surprising given that cg19610905 is located downstream of *FADS2*. Gene expression of *TMEM258* in whole blood was negatively associated with DNA methylation at cg19610905. The directionality test suggested that DNA methylation influences TMEM258 expression at this locus rather than the opposite direction of effect (*P* < 1 × 10^−16^).

We did not identify evidence in the colocalization analysis suggesting that DNA methylation plays a role in trait variation at the *SORT1* region. However, there was evidence of tissue specificity in liver tissue which supports the evidence identified in our MR analysis. The first plot in Fig. 3b illustrates how the effects on *SORT1* gene expression and total cholesterol at this region colocalizes in liver tissue. In contrast, the neighbouring plot depicts the same analysis but in whole blood, whereby no evidence of colocalization was detected. Furthermore, we see the same tissue-specific colocalization for the effect on ApoB in the same region (Additional file 2: Table S17). The *CELSR2* gene showed similar evidence for tissue specificity in the liver, whereas *PSRC1* expression colocalized with GWAS traits in both the whole blood and liver.

### Genome-wide application using findings from large-scale consortia

**Fig. 4:**
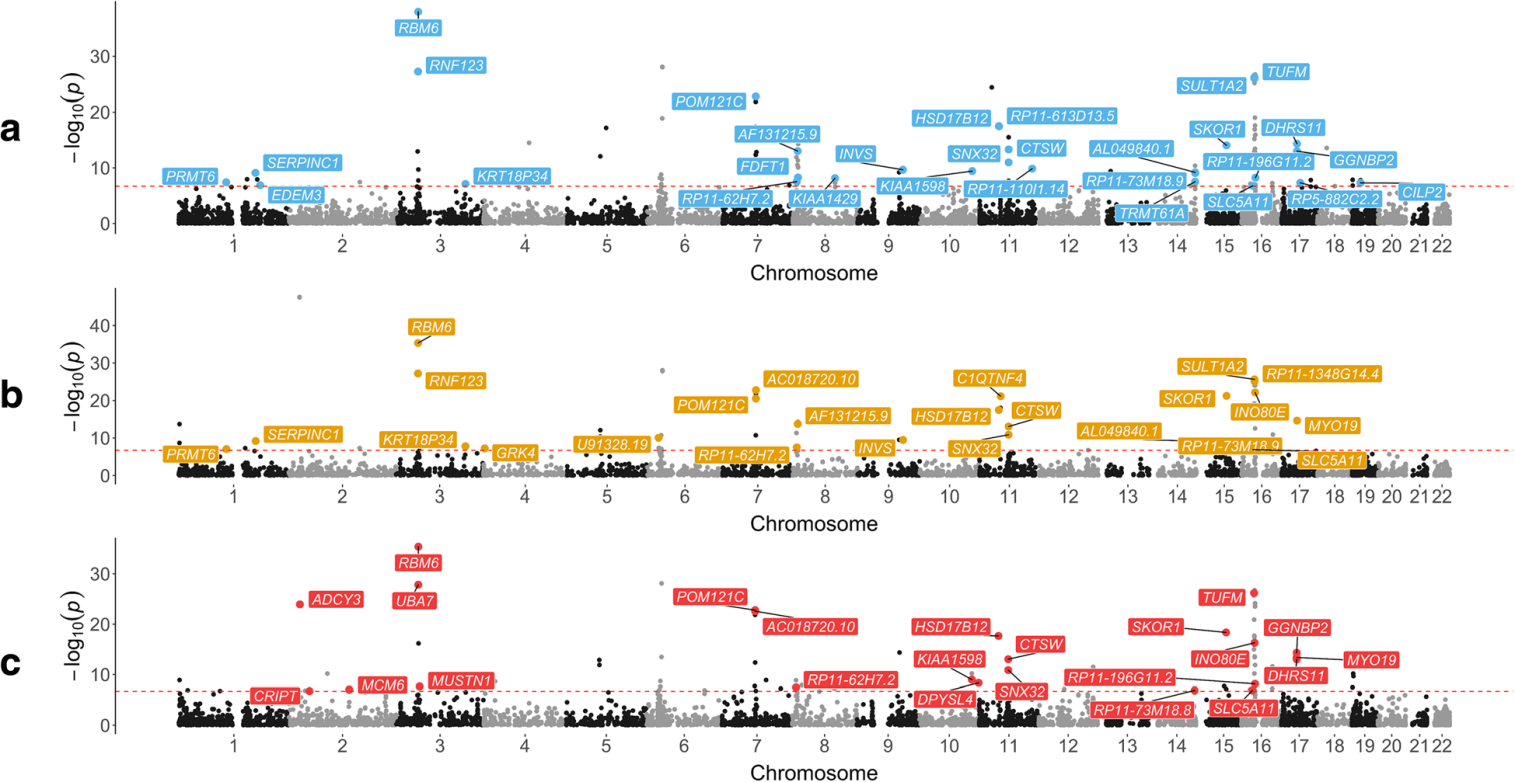
Manhattan plots illustrating associations with body mass index (BMI) across three different tissue types from Mendelian randomization analyses: adipose subcutaneous (**a**), adipose visceral omentum (**b**) and whole blood (**c**). Chromosomal position of genetic variants used as instrumental variables is plotted on the *x*-axis against −log10 *P* values from the Wald ratio on the *y*-axis. Associations that survived the multiple testing threshold (1.9 × 10^−7^—represented by the red horizontal line) are coloured according to the tissue type used

Applying our pipeline using publicly available GWAS findings involved 256,011 tests between eQTL across 9 tissue types and 7 cardiovascular traits. Overall, 233 associations (across 80 unique genes) survived multiple testing corrections (*P* < 1.9 × 10^−07^ (0.05/256,011)) that also provided evidence of colocalization (i.e. PPA ≥ 0.8). This suggests that gene expression and cardiovascular traits share a causal variant at their loci (Additional file 2: Table S22). Furthermore, 156 of the 233 associations which colocalized provided evidence that the underlying causal variants at their loci may also influence nearby DNA methylation levels (PPA ≥ 0.8 for scenario GEM). Figure 4 contains Manhattan plots illustrating the associations with BMI across 3 different tissue types (adipose–subcutaneous; adipose–visceral omentum; and whole blood).

Investigating the findings with BMI suggests that there are loci which are most likely attributed to scenario 3 over alternative explanations, given that they were the only genes in their region which provided evidence of colocalization. An example of this is *SLC5A11* which was associated with BMI using gene expression from six different tissue types (all PPA > 0.90). There were also effects which may be attributed to scenario 2 that appear to be more tissue-specific, such as *POMC*, where evidence of association was only detected using data derived from pancreatic tissue (PPA = 0.92). Furthermore, these analyses may help elucidate novel loci in disease risk, such as the *CILP2* gene (PPA = 0.83). The GWAS SNP at this locus does not meet conventional GWAS corrections (*P* = 7.16 × 10^−08^), although the reduced burden of multiple testing by integrating tissue-specific expression data strengthens the evidence that it may influence disease risk. However, there were also loci where multiple genes provided evidence of colocalization, such as *TUFM* and *SULT1A2* located on chromosome 16 (PPA = 0.93 and 0.99 respectively). Further analyses are therefore necessary to help distinguish whether there are multiple causal genes at these loci (i.e. scenario 2) or whether a single causal gene is responsible for the association signal (scenario 3).

## Discussion

In this study, we have developed an analysis pipeline to elucidate transcriptional mechanisms in disease which can help explain the functional relevance of GWAS findings. This is achieved by adapting the principles of MR in evaluating the putative effect of tissue-specific gene expression on complex traits, which can be complemented with moloc and harnessing large-scale summary statistics. We demonstrate the value of this approach by evaluating 11 signals identified in an eQTLWAS study undertaken in a cohort of young individuals from ALSPAC. Tissue-specific analyses helped infer whether individual or multiple genes were potentially responsible for observed signals at each locus. Furthermore, moloc suggested that changes in gene expression and proximal DNA methylation may influence disease susceptibility at the *FADS1* locus.

The *ADCY3* locus has been reported to be associated with BMI in young individuals in previous studies [48, 49]. Our MR analyses identified evidence that changes in *ADCY3* expression in adipose tissues may influence BMI, whereas weaker evidence was observed based on the expression of other proximal genes (such as *NCOA1*). Specifically, we found that the magnitude of the effect for *ADCY3* expression was observed most strongly in adipose tissue, aligning with other research [50, 51]. Furthermore, recent work has uncovered loss-of-function variants in *ADCY3* associated with an increase in obesity levels [52]. In contrast, moloc showed a lack of evidence of colocalization for *NCOA1* expression. Moreover, although the *CENPO* gene was evaluated as part of our original association analysis, there were no eQTLs for this gene for any of the tissues we analysed. From this, we believe that *ADCY3* is likely the functional gene impacting BMI at this locus (Fig. 1a: scenario 3), although only with in-depth follow-up analyses can this be determined with confidence. Our additional analysis indicated no tissue-specific effects using eQTL effect estimates derived from brain tissue, which suggests that the influence of *ADCY3* expression on BMI levels may be confined to adipose tissue. However, extended analyses using molecular data derived from brain tissue are necessary to confirm this, particularly given that previous work has linked gene expression in brain tissue with obesity-related traits [50, 53].

We also identified evidence of colocalization for gene expression, DNA methylation and complex trait variation at the cholesterol-associated region on chromosome 11. This was observed for *TMEM258* expression in whole blood, although *FADS1* narrowly missed the 0.8 cut-off (PPA = 0.77). This was based on DNA methylation levels at a CpG site located in the promoter region of *FADS1* (cg19610905). This effect was observed using data from whole blood (which is the only tissue we had accessible DNA methylation for in this study), which is potentially acting as a proxy for the true causal/relevant tissue type for this effect [18]. However, there was no indication that methylation played a role in the expression of *FADS2*. *TMEM258* has been proposed as a regulatory site for cholesterol in ‘abdominal fat’ previously [54]. There is also previously evidence of association between FADS1 and cholesterol levels in young individuals [55]. Additionally, genetic variation at this region is associated with DNA methylation levels at cg19610905 based on cord blood in ARIES, which suggests that these methylation changes may influence the expression of *FADS1/TMEM258* from a very early age. Overall at this region, our results suggest that scenario 2 (Fig. 1a) is a likely explanation for the association signal, where it is biologically plausible that multiple causal genes influence complex trait variation. Specifically, our analyses suggest that *TMEM258* and *FADS1* are potential causal genes; however, further work is needed to elucidate whether *FADS2* is directly influencing cardiovascular traits or is simply co-regulated with nearby functional loci.

The *LPL* locus was not co-regulated with other genes in our study and is therefore likely attributed to scenario 1 (Fig. 1a). *LPL* has been previously reported to influence lipid and triglyceride levels [56–58] which is supported by evidence from gene knockout experiments [59]. The tissue specificity of *LPL* has also previously been explored, although not by recent studies [60]. Two-sample MR analyses provided robust evidence that this gene is strongly expressed in adipose tissue which corroborates previous research [60, 61].

At other associated loci, the *SORT1* locus has been previously studied in detail with regard to its effect on cholesterol levels [46, 62]. Our MR analyses provided additional evidence of an effect using expression derived from liver tissue for *SORT1*, *CELSR2* and *PSRC1*, as well as in pancreatic tissue for *SORT1* and *CELSR2* only. Our subsequent moloc analysis identified evidence of colocalization for *SORT1* and *CELSR2* expression with cholesterol only in liver tissue, suggesting that *PSRC1* could be less tissue-specific than the other two genes in this region. Previous research supports these observations with regard to the effects of *SORT1* and *CELSR2* in the liver [11, 63], as well as the lack of tissue specificity for the *PSRC1* locus [64]. There was limited evidence that variation in this region colocalized with DNA methylation, although future work with methylation data derived from liver tissue is warranted.

Extending our analysis pipeline to leverage summary-level data from large-scale consortia identified a total of 233 association signals (220 excluding previous findings in this study). Additionally, there was evidence that genetic variants at these loci may also influence nearby DNA methylation levels for 156 of these effects. As our methylation data was derived from whole blood, these findings support recent reports that whole blood may act as a reliable proxy for other tissue types [21]. These results can be harnessed to prioritize candidate genes which may be responsible for the associations detected by GWAS. Amongst findings, we identified evidence of association at known loci which influence cardiovascular and anthropometric traits, such as *SLC5A11*, *SERPINC1* and *INO80E* [65]. There was also evidence supporting potentially novel loci, where findings from GWAS alone would not survive multiple testing corrections. An example of this is *CILP2*, a carbohydrate binding gene, which was associated with BMI in our analysis.

There are also examples indicating that the integration of tissue-specific gene expression can help develop understanding into the biological mechanisms by which these genes influence cardiovascular traits. For instance, we identified evidence supporting *POMC* as responsible for the association with BMI at its locus which was only detected using pancreatic tissue data (PPA = 0.92). Therefore, the most parsimonious explanation for this finding may be that variation at this locus influences insulin regulation via melanocortin peptides which are derived from pro-opiomelanocortins (the product of *POMC*) [66]. This finding also supports the evidence that melanocortins play a role in insulin regulation within the pancreas previously demonstrated in animal models [67]. Furthermore, our colocalization results indicate that the most likely scenario at this locus suggests that DNA methylation may play a role in this effect (PPA = 0.79), supporting previous findings that hypermethylation of *POMC* influences obesity risk [68].

This study has demonstrated the value of our analysis pipeline in terms of distinguishing between scenarios 1, 2, 3 and 4 (Fig. 1a). However, an important limiting factor, as with any study applying single-instrument MR, is the inability to separate mediation from horizontal pleiotropy (i.e. scenario 5). Given that trans-eQTLs likely regulate genes through a non-allele-specific mechanism [69], we selected only eQTLs that were influencing proximal genes. This is also a limitation of alternative approaches which are comparable to ours, such as the SMR and eCAVIAR methods [70, 71]. These methods have advantages over our approach, for example, eCAVIAR allows multiple causal variants to be identified in a region of interest, whereas SMR routinely implements its colocalization approach (known as HEIDI) to help identify regions with shared causal variants between traits. However, our approach also has advantages, such as investigating shared causal variants for more than two traits (e.g. cardiovascular traits, gene expression and DNA methylation in this study), whereas these alternatives are currently confined to looking at a maximum of two traits. As more eQTLs are uncovered across the genome by future studies, across a wide range of tissue and cell types, our analysis pipeline and other similar approaches should become increasingly powerful to evaluate all five outlined scenarios.

In terms of limitations in this study, we recognize that the varying sample sizes between tissues in GTEx will determine the relative power to detect eQTL (Additional file 1: Fig. S3). Increased sample sizes in GTEx [72] and similar endeavours will help address this limitation. Furthermore, the DNA methylation data we incorporated within our pipeline from the accessible resource for ARIES [45] project was only obtained in whole blood. This is a limitation as it relies on the assumption that whole blood is acting as a proxy for another more relevant tissue type to the disease of interest [73]. Although our genome-wide results support recent findings indicating that this assumption often holds, there are also potentially many loci where this is not the case. As such, future work will need to incorporate DNA methylation data from various tissues as and when these data become available to help better understand the role of this epigenetic process on transcriptional activity. This is a particularly important question given recent evidence suggesting that promoter DNA methylation may not be sufficient on its own to influence transcriptional changes [74]. Therefore, a resource concerning tissue-specific DNA methylation would be extremely valuable.

Another constraint of relatively modest sample sizes in GTEx is that we did not detect evidence of colocalization at some loci despite investigating the functionally relevant gene. For example, we can be reasonably certain that circulating ApoA1 levels are influenced by the expression of *APOA1*. The complexity of gene regulation is often underestimated due to factors such as feedback loops, hidden confounders in expression data and regulatory activity not always being detected in relevant tissues [75]. However, we are beginning to better understand the regulation across tissues [64], which should provide us with further opportunities to detect cross-tissue regulatory activity and develop our biological understanding of a disease. We also note that fine-mapping and colocalization approaches may be limited in their inference when applied to the regions of the genome with extensive linkage disequilibrium, such as the HLA region of the human genome. As such, findings at these loci should be interpreted with caution, particularly when evaluating association signals that exist between multiple correlated traits such as the effect of *SORT1* in Additional file 2: Table S5 of our study.

## Conclusions

We have identified a number of tissue-specific effects at several regions throughout the genome. Our results suggest that DNA methylation may also influence complex traits through gene expression pathways. In-depth evaluations of the loci identified in our study should help to fully understand the causal pathway to disease for these effects. Furthermore, as these genetic loci may influence cardiovascular traits early in the life course, these endeavours should allow a long window of intervention for disease susceptibility. Finally, the analysis pipeline outlined in this study should prove particularly valuable for future studies as increasingly large datasets concerning tissue-specific gene expression become available.

## Additional files

Additional file 1: **Fig. S1.** MR effect estimates are based on the Wald ratio test, where ***β***^^^***Y***|***Z*** is the coefficient of the genetic variant in the regression of the exposure (e.g. gene expression) and ***β***^^^***Y***|***Z*** is the coefficient of the genetic variant in the regression of the outcome (e.g. cardiovascular trait). **Fig. S2.** Volcano plots illustrating tissue-specific MR results. Effect sizes and *P* values obtained from the MR Wald ratio. **Fig. S3.** Scatter plot illustrating how eGene discovery increases as sample size increases (*R*^2^ = 0.84). Figure adapted from the Genotype Tissue Expression Project [15]. **Fig. S4.** Volcano plot from our tissue-specific Mendelian randomization analysis for the apolipoprotein A1-associated region (rs2727784). Outcome data from [39].** Fig. S5.** Volcano plot from our tissue-specific Mendelian randomization analysis for the apolipoprotein B-associated region (rs646776). Outcome data from [39]. **Fig. S6.** Volcano plot from our tissue-specific Mendelian randomization analysis for the apolipoprotein B-associated region (rs10419998). Outcome data from [39]. **Fig. S7.** Volcano plot from our tissue-specific Mendelian randomization analysis for the cholesterol-associated region (rs646776). Outcome data from Willer et al. (2016). **Fig. S8.** Volcano plot from our tissue-specific Mendelian randomization analysis for the low-density lipoprotein-associated region (rs646776). Outcome data from Willer et al. (2016). **Fig. S9.** Volcano plot from our tissue-specific Mendelian randomization analysis for the triglyceride-associated region (rs80026582). Outcome data from Willer et al. (2016). **Fig. S10.** Volcano plot from our tissue-specific Mendelian randomization analysis for the very low-density lipoprotein-associated region (rs80026582). Outcome data from [39]. (PDF 1036 kb)

Additional file 2: **Table S1.** Tissues used for tissue-specific Mendelian randomization. **Table S2.** Details on the GWAS datasets used. **Table S3.** Explanation of the 15 possible scenarios summarizing how variants how can be shared amongst three traits. **Table S4.** Tissues used for moloc analysis. **Table S5.** Results of fine-mapping analysis using FINEMAP. **Table S6.** Tissue-specific Mendelian randomization results for the apoliporotein A1-associated region on chromosone 11 (rs2727784). **Table S7.** Tissue-specific Mendelian randomization results for the apolipoprotein B-associated region on chromosone 1 (rs646776). **Table S8.** Tissue-specific Mendelian randomization results for the apolipoprotein B-associated region on chromosone 19 (rs10419998). **Table S9.** Tissue-specific Mendelian randomization results for the body mass index-associated region on chromosone 2 (rs11693654). **Table S10.** Tissue-specific Mendelian randomization results for the cholesterol-associated region on chromosone 1 (rs646776). **Table S11.** Tissue-specific Mendelian randomization results for the cholesterol-associated region on chromosone 11 (rs174538). **Table S12.** Tissue-specific Mendelian randomization results for the interleukin-6-associated region on chromosone 1 (rs12129500). **Table S13.** Tissue-specific Mendelian randomization results for the interleukin-6-associated region on chromosone 9 (rs600038). **Table S14.** Tissue-specific Mendelian randomization results for the low-density lipoprotein-associated region on chromosone 1 (rs646776). **Table S15.** Tissue-specific Mendelian randomization results for the triglyceride-associated region on chromosone 8 (rs80026582). **Table S16.** Tissue-specific Mendelian randomization results for the very low-density lipoprotein-associated region on chromosone 8 (rs80026582). **Table S17.** moloc results for the apolipoprotein B-associated region on chromosone 1. **Table S18.** moloc results for the cholesterol-associated region on chromosone 1. **Table S19.** moloc results for the body mass index-associated region on chromosone 2. **Table S20.** moloc results for the cholesterol-associated region on chromosone 11. **Table S21.** moloc results for the low-density lipoprotein-associated region on chromosone 1. **Table S22.** Top findings from applying our pipeline genome-wide for seven cardiovascular traits from GWAS consortia across nine different tissue types. (XLSX 136 kb)

## Abbreviations

ALSPAC: Avon Longitudinal Study of Parents and Children
ApoA1: Apolipoprotein A1
ApoB: Apolipoprotein B
ARIES: Accessible Resource for Integrated Epigenomic Studies
BMI: Body mass index
CRP: C-reactive protein
eQTL: Expression quantitative trait loci
eQTLWAS: eQTL-wide association study
GTEx: Genotype Tissue Expression Project
GWAS: Genome-wide association study
IL-6: Interleukin 6
LD: Linkage disequilibrium
LDL: Low-density lipoprotein
MAF: Minor allele frequency
Mb: Megabase
moloc: Multiple-trait colocalization
mQTL: Methylation quantitative trait loci
MR: Mendelian randomization
mRNA: Messenger ribonucleic acid
PPA: Posterior probability of association
SNP: Single nucleotide polymorphism
TSS: Transcription start site
TWAS: Transcriptome-wide association study
VLDL: Very low-density lipoprotein

## References

[1] World Health Organization. Cardiovascular disease: global atlas on cardiovascular disease prevention and control. Geneva: World Health Organization; 2012.

[2] Altshuler D, Daly MJ, Lander E (2009). Genetic mapping in human disease. Science (80- ).

[3] Smith JG, Newton-Cheh C. Genome-wide association studies of late-onset cardiovascular disease. J. Mol. Cell. Cardiol. 2015;83:131–41. 10.1016/j.yjmcc.2015.04.004 PMC4459905 25870159

[4] Holmes MV, Asselbergs FW, Palmer TM, Drenos F, Lanktree MB, Nelson CP (2015). Mendelian randomization of blood lipids for coronary heart disease. Eur Heart J.

[5] Mihaylova B, Emberson J, Blackwell L, Keech A, Simes J, Barnes EH, et al. The effects of lowering LDL cholesterol with statin therapy in people at low risk of vascular disease: meta-analysis of individual data from 27 randomised trials. Lancet [Internet]. 2012;380:581–590. Available from: http://www.pubmedcentral.nih.gov/articlerender.fcgi?artid=3437972&tool=pmcentrez&rendertype=abstract%0A, http://www.sciencedirect.com/science/article/pii/S0140673612603675 10.1016/S0140-6736(12)60367-5 PMC3437972 22607822

[6] Hindorff LA, Sethupathy P, Junkins HA, Ramos EM, Mehta JP, Collins FS (2009). Potential etiologic and functional implications of genome-wide association loci for human diseases and traits. Proc Natl Acad Sci [Internet].

[7] Edwards SL, Beesley J, French JD, Dunning M. Beyond GWASs: illuminating the dark road from association to function. Am. J. Hum. Genet. 2013:779–97. 10.1016/j.ajhg.2013.10.012 PMC3824120 24210251

[8] Joehanes R, Zhang X, Huan T, Yao C, Ying S, Nguyen QT, et al. Integrated genome-wide analysis of expression quantitative trait loci aids interpretation of genomic association studies. Genome Biol [Internet]. 2017;18:16. Available from: https://genomebiology.biomedcentral.com/articles/10.1186/s13059-016-1142-6 10.1186/s13059-016-1142-6 PMC5264466 28122634

[9] Gusev A, Ko A, Shi H, Bhatia G, Chung W, Penninx BWJH, et al. Integrative approaches for large-scale transcriptome-wide association studies. Nat Genet [Internet]. Nature Publishing Group; 2016;48:245–252. Available from: http://www.nature.com/doifinder/10.1038/ng.3506 10.1038/ng.3506 PMC4767558 26854917

[10] Nica AC, Dermitzakis ET. Expression quantitative trait loci: present and future. Philos Trans R Soc Lond B Biol Sci [Internet]. 2013;368:20120362. Available from: http://www.ncbi.nlm.nih.gov/pubmed/23650636%5Cn 10.1098/rstb.2012.0362 PMC3682727 23650636

[11] Wainberg M, Sinnott-Armstrong N, Knowles D, Golan D, Ermel R, Ruusalepp A, et al. Vulnerabilities of transcriptome-wide association studies. bioRxiv [Internet]. 2017; Available from: http://biorxiv.org/content/early/2017/10/20/206961.abstract 10.1038/s41588-019-0385-z PMC6777347 30926968

[12] Davey Smith G, Hemani G. Mendelian randomization: genetic anchors for causal inference in epidemiological studies. Hum Mol Genet [Internet]. 2014;23:R89–98. Available from: http://www.ncbi.nlm.nih.gov/pubmed/25064373%5Cn. 10.1093/hmg/ddu328 PMC4170722 25064373

[13] Davey Smith G, Ebrahim S (2003). “Mendelian randomization”: can genetic epidemiology contribute to understanding environmental determinants of disease?. Int J Epidemiol.

[14] Lawlor DA (2016). Commentary: two-sample Mendelian randomization: opportunities and challenges. Int J Epidemiol [Internet].

[15] Aguet F, Ardlie KG, Cummings BB, Gelfand ET, Getz G, Hadley K (2017). Genetic effects on gene expression across human tissues. Nature [Internet]..

[16] Giambartolomei C, Zhenli Liu J, Zhang W, Hauberg M, Shi H, Boocock J, et al. A Bayesian framework for multiple trait colocalization from summary association statistics. Bioinformatics [Internet]. 2018; Available from: https://www.ncbi.nlm.nih.gov/pubmed/29579179. 10.1093/bioinformatics/bty147 PMC6061859 29579179

[17] Wahl S, Drong A, Lehne B, Loh M, Scott WR, Kunze S (2016). Epigenome-wide association study of body mass index, and the adverse outcomes of adiposity. Nature [Internet].

[18] Qi T, Wu Y, Zeng J, Zhang F, Xue A, Jiang L, et al. Identifying gene targets for brain-related traits using transcriptomic and methylomic data from blood. Nat Commun. 2018;9:1–26. 10.1038/s41467-018-04558-1 PMC5995828 29891976

[19] Bonder MJ, Luijk R, Zhernakova DV, Moed M, Deelen P, Vermaat M (2017). Disease variants alter transcription factor levels and methylation of their binding sites. Nat Genet.

[20] Acharya CR, Owzar K, Allen AS. Mapping eQTL by leveraging multiple tissues and DNA methylation. BMC Bioinformatics. 2017;18:1856–59 10.1186/s12859-017-1856-9 PMC5648503 29047346

[21] Hannon E, Dempster E, Viana J, Burrage J, Smith AR, Macdonald R, et al. An integrated genetic-epigenetic analysis of schizophrenia: evidence for co-localization of genetic associations and differential DNA methylation. Genome Biol. 2016;17:176. 10.1186/s13059-016-1041-x PMC5004279 27572077

[22] Hodgkin J. Seven types of pleiotropy. Int. J. Dev. Biol. 1998;42:501–5. 9654038

[23] Golding J, Pembrey M, Jones R (2001). ALSPAC--the Avon Longitudinal Study of Parents and Children. I. Study methodology. Paediatr Perinat Epidemiol.

[24] Fraser A, Macdonald-wallis C, Tilling K, Boyd A, Golding J, Davey Smith G (2013). Cohort profile: the Avon Longitudinal Study of Parents and Children: ALSPAC mothers cohort. Int J Epidemiol.

[25] Boyd A, Golding J, Macleod J, Lawlor DA, Fraser A, Henderson J (2013). Cohort profile: the ‘children of the 90s’-the index offspring of the Avon Longitudinal Study of Parents and Children. Int J Epidemiol.

[26] University of Bristol. Accessing the resource [Internet]. [cited 2018 Jan 29]. Available from: http://www.bristol.ac.uk/alspac/researchers/access/

[27] 1000 Genomes Project Consortium, Auton A, Brooks LD, Durbin RM, Garrison EP, Kang HM, et al. A global reference for human genetic variation. Nature [Internet]. 2015;526:68–74. Available from: https://www.nature.com/articles/nature15393. 10.1038/nature15393 PMC4750478 26432245

[28] McCarthy S, Das S, Kretzschmar W, Delaneau O, Wood AR, Teumer A (2016). A reference panel of 64,976 haplotypes for genotype imputation. Nat Genet.

[29] Warnick GR, Knopp RH, Fitzpatrick V, Branson L (1990). Estimating low-density lipoprotein cholesterol by the Friedewald equation is adequate for classifying patients on the basis of nationally recommended cutpoints. Clin Chem.

[30] Falaschetti E, Hingorani AD, Jones A, Charakida M, Finer N, Whincup P (2010). Adiposity and cardiovascular risk factors in a large contemporary population of pre-pubertal children. Eur Heart J.

[31] Westra HJ, Peters MJ, Esko T, Yaghootkar H, Schurmann C, Kettunen J (2013). Systematic identification of trans eQTLs as putative drivers of known disease associations. Nat Genet.

[32] Conesa A, Madrigal P, Tarazona S, Gomez-Cabrero D, Cervera A, McPherson A, et al. A survey of best practices for RNA-seq data analysis. Genome Biol. 2016. 10.1186/s13059-016-0881-8 PMC4728800 26813401

[33] Chang CC, Chow CC, Tellier LCAM, Vattikuti S, Purcell SM, Lee JJ. Second-generation PLINK: rising to the challenge of larger and richer datasets. Gigascience. 2015;4:47-48. 10.1186/s13742-015-0047-8 PMC4342193 25722852

[34] Purcell S, Chang C. PLINK 1.9 [Internet]. 2015 [cited 2018 Jan 9]. Available from: www.cog-genomics.org/plink/1.9/

[35] Turner SD. qqman: an R package for visualizing GWAS results using Q-Q and Manhattan plots [Internet]. bioRxiv. 2014. Available from: http://biorxiv.org/lookup/doi/10.1101/005165

[36] Benner C, Spencer CCA, Havulinna AS, Salomaa V, Ripatti S, Pirinen M (2016). FINEMAP: efficient variable selection using summary data from genome-wide association studies. Bioinformatics.

[37] Benner C, Havulinna AS, Järvelin MR, Salomaa V, Ripatti S, Pirinen M. Prospects of fine-mapping trait-associated genomic regions by using summary statistics from genome-wide association studies. Am J Hum Genet. 2017;101. 10.1016/j.ajhg.2017.08.012 PMC5630179 28942963

[38] Burgess S, Small DS, Thompson SG. A review of instrumental variable estimators for Mendelian randomization. Stat Methods Med Res. 2017;26. 10.1177/0962280215597579 PMC5642006 26282889

[39] Kettunen J, Demirkan A, Würtz P, Draisma HHM, Haller T, Rawal R, et al. Genome-wide study for circulating metabolites identifies 62 loci and reveals novel systemic effects of LPA. Nat Commun. 2016;7:11122. 10.1038/ncomms11122 PMC4814583 27005778

[40] Willer CJ, Schmidt EM, Sengupta S, Peloso GM, Gustafsson S, Kanoni S (2013). Discovery and refinement of loci associated with lipid levels. Nat Genet.

[41] Bycroft C, Freeman C, Petkova D, Band G, Elliott LT, Sharp K, et al. The UK Biobank resource with deep phenotyping and genomic data. Nature [Internet]. 2018; Available from: https://www.nature.com/articles/s41586-018-0579-z 10.1038/s41586-018-0579-z PMC6786975 30305743

[42] Hemani G, Zheng J, Wade KH, Laurin C, Elsworth B, Burgess S, et al. The MR-base platform supports systematic causal inference across the human phenome. Elife [Internet]. 2018; Available from: https://elifesciences.org/articles/34408. 10.7554/eLife.34408 PMC5976434 29846171

[43] Wickham H. ggplot2 elegant graphics for data analysis [Internet]. Media. 2009. Available from: https://cran.r-project.org/web/packages/ggplot2/index.html

[44] Hemani G, Tilling K, Davey SG. Orienting the causal relationship between imprecisely measured traits using GWAS summary data. PLoS Genet. 2017;13. 10.1371/journal.pgen.1007081 PMC5711033 29149188

[45] Relton CL, Gaunt T, McArdle W, Ho K, Duggirala A, Shihab H (2015). Data resource profile: Accessible Resource For Integrated Epigenomic Studies (ARIES). Int J Epidemiol.

[46] Musunuru K, Strong A, Frank-Kamenetsky M, Lee NE, Ahfeldt T, Sachs KV (2010). From noncoding variant to phenotype via SORT1 at the 1p13 cholesterol locus. Nature.

[47] Pickrell JK, Berisa T, Liu JZ, Ségurel L, Tung JY, Hinds DA (2016). Detection and interpretation of shared genetic influences on 42 human traits. Nat Genet.

[48] Stergiakouli E, Gaillard R, Tavaré JM, Balthasar N, Loos RJ, Taal HR (2014). Genome-wide association study of height-adjusted BMI in childhood identifies functional variant in ADCY3. Obesity.

[49] Namjou B, Keddache M, Marsolo K, Wagner M, Lingren T, Cobb B, et al. EMR-linked GWAS study: investigation of variation landscape of loci for body mass index in children. Front Genet. 2013;4:268. 10.3389/fgene.2013.00268 PMC3847941 24348519

[50] Hao R-H, Yang T-L, Rong Y, Yao S, Dong S-S, Chen H, et al. Gene expression profiles indicate tissue-specific obesity regulation changes and strong obesity relevant tissues. Int J Obes [Internet]. 2018:1–7 Available from: http://www.nature.com/doifinder/10.1038/ijo.2017.283. 10.1038/ijo.2017.283 29151593

[51] Vink RG, Roumans NJ, Fazelzadeh P, Tareen SH, Boekschoten MV, van Baak MA (2017). Adipose tissue gene expression is differentially regulated with different rates of weight loss in overweight and obese humans. Int J Obes [Internet].

[52] Grarup N, Moltke I, Andersen MK, Dalby M, Vitting-Seerup K, Kern T, et al. Loss-of-function variants in ADCY3 increase risk of obesity and type 2 diabetes. Nat Genet. 2018;50. 10.1038/s41588-017-0022-7 PMC5828106 29311636

[53] Samad F, Pandey M, Loskutoff DJ (2001). Regulation of tissue factor gene expression in obesity. Blood.

[54] Franzén O, Ermel R, Cohain A, Akers NK, Di Narzo A, Talukdar HA (2016). Cardiometabolic risk loci share downstream cis- and trans-gene regulation across tissues and diseases. Science (80- ).

[55] Dumont J, Huybrechts I, Spinneker A, Gottrand F, Grammatikaki E, Bevilacqua N (2011). FADS1 genetic variability interacts with dietary-linolenic acid intake to affect serum non-HDL-cholesterol concentrations in European adolescents. J Nutr [Internet].

[56] Johansen CT, Kathiresan S, Hegele RA (2011). Genetic determinants of plasma triglycerides. J Lipid Res [Internet].

[57] Humphries SE, Nicaud V, Margalef J, Tiret L, Talmud PJ (1998). Lipoprotein lipase gene variation is associated with a paternal history of premature coronary artery disease and fasting and postprandial plasma triglycerides: the European Atherosclerosis Research Study (EARS). Arterioscler Thromb Vasc Biol.

[58] Mead JR, Irvine Sa, Ramji DP. Lipoprotein lipase: structure, function, regulation, and role in disease. J Mol Med (Berl) [Internet]. 2002;80:753–769. Available from: http://www.ncbi.nlm.nih.gov/pubmed/12483461. 10.1007/s00109-002-0384-9 12483461

[59] Chen Y, Zhu J, Lum PY, Yang X, Pinto S, MacNeil DJ (2008). Variations in DNA elucidate molecular networks that cause disease. Nature.

[60] Ranganathan G, Ong JM, Yukht A, Saghizadeh M, Simsolo RB, Pauer A (1995). Tissue-specific expression of human lipoprotein lipase: effect of the 3′-untranslated region on translation. J Biol Chem.

[61] Wang H, Eckel RH (2009). Lipoprotein lipase: from gene to obesity. Am J Physiol Endocrinol Metab [Internet].

[62] Arvind P, Nair J, Jambunathan S, Kakkar VV, Shanker J (2014). CELSR2-PSRC1-SORT1 gene expression and association with coronary artery disease and plasma lipid levels in an Asian Indian cohort. J Cardiol.

[63] Schadt EE, Molony C, Chudin E, Hao K, Yang X, Lum PY (2008). Mapping the genetic architecture of gene expression in human liver. PLoS Biol.

[64] Ongen H, Brown AA, Delaneau O, Panousis NI, Nica AC, Dermitzakis ET (2017). Estimating the causal tissues for complex traits and diseases. Nat Genet.

[65] Hu B, Wang Q, Tang L, Hu Y, Liu H. A predominant mutation in regulatory region of SERPINC1 gene and venous thrombosis. Blood [Internet]. 2015;126:4669 LP-4669. Available from: http://www.bloodjournal.org/content/126/23/4669.abstract

[66] Gantz I, Fong TM (2003). The melanocortin system. Am J Physiol Endocrinol Metab.

[67] Mansour M, White D, Wernette C, Dennis J, Tao YX, Collins R (2010). Pancreatic neuronal melanocortin-4 receptor modulates serum insulin levels independent of leptin receptor. Endocrine.

[68] Kuehnen P, Mischke M, Wiegand S, Sers C, Horsthemke B, Lau S, et al. An alu element-associated hypermethylation variant of the POMC gene is associated with childhood obesity. PLoS Genet. 2012. 10.1371/journal.pgen.1002543 PMC3305357 22438814

[69] Albert FW, Kruglyak L. The role of regulatory variation in complex traits and disease. Nat. Rev. Genet. 2015;16:197–212. 10.1038/nrg3891 25707927

[70] Zhu Z, Zhang F, Hu H, Bakshi A, Robinson MR, Powell JE (2016). Integration of summary data from GWAS and eQTL studies predicts complex trait gene targets. Nat Genet [Internet].

[71] Hormozdiari F, van de Bunt M, Segrè AV, Li X, Joo JWJ, Bilow M, et al. Colocalization of GWAS and eQTL signals detects target genes. Am J Hum Genet. 2016;99. 10.1016/j.ajhg.2016.10.003 PMC5142122 27866706

[72] Stranger BE, Brigham LE, Hasz R, Hunter M, Johns C, Johnson M, et al. Enhancing GTEx by bridging the gaps between genotype, gene expression, and disease. Nat. Genet. 2017;49:1664–70. 10.1038/ng.3969 PMC6636856 29019975

[73] Teschendorff AE, Relton CL. Statistical and integrative system-level analysis of DNA methylation data. Nat Rev Genet [Internet]. 2017; Available from: http://www.nature.com/doifinder/10.1038/nrg.2017.86. 10.1038/nrg.2017.86 29129922

[74] Ford EE, Grimmer MR, Stolzenburg S, Bogdanovic O, de Mendoza A, Farnham PJ, et al. Frequent lack of repressive capacity of promoter DNA methylation identified through genome-wide epigenomic manipulation. bioRxiv [Internet]. 2017; Available from: http://biorxiv.org/content/early/2017/09/20/170506.abstract.

[75] Torres JM, Barbeira AN, Bonazzola R, Morris AP, Shah KP, Wheeler HE, et al. Integrative cross tissue analysis of gene expression identifies novel type 2 diabetes genes. bioRxiv [Internet]. 2017;108134. Available from: http://biorxiv.org/content/early/2017/02/27/108134

