# Supplementary material for: Prioritizing putative influential genes in cardiovascular disease susceptibility by applying tissue-specific Mendelian randomization
Source: Genome Med. 2019 Jan 31;11:6. doi: 10.1186/s13073-019-0613-2 (PMC6354354; doi:10.1186/s13073-019-0613-2)
Supplement: Supplementary file 1 — Fig. S1. MR effect estimates are based on the Wald ratio test, where β^Y|Z is the coefficient of the genetic variant in the regression of the exposure (e.g. gene expression) and β^Y|Z is the coefficient of the genetic variant in the regression of the outcome (e.g. cardiovascular trait). Fig. S2. Volcano plots illustrating tissue-specific MR results. Effect sizes and P values obtained from the MR Wald ratio. Fig. S3. Scatter plot illustrating how eGene discovery increases as sample size increases (R2 = 0.84). Figure adapted from the Genotype Tissue Expression Project [15]. Fig. S4. Volcano plot from our tissue-specific Mendelian randomization analysis for the apolipoprotein A1-associated region (rs2727784). Outcome data from [39]. Fig. S5. Volcano plot from our tissue-specific Mendelian randomization analysis for the apolipoprotein B-associated region (rs646776). Outcome data from [39]. Fig. S6. Volcano plot from our tissue-specific Mendelian randomization analysis for the apolipoprotein B-associated region (rs10419998). Outcome data from [39]. Fig. S7. Volcano plot from our tissue-specific Mendelian randomization analysis for the cholesterol-associated region (rs646776). Outcome data from Willer et al. (2016). Fig. S8. Volcano plot from our tissue-specific Mendelian randomization analysis for the low-density lipoprotein-associated region (rs646776). Outcome data from Willer et al. (2016). Fig. S9. Volcano plot from our tissue-specific Mendelian randomization analysis for the triglyceride-associated region (rs80026582). Outcome data from Willer et al. (2016). Fig. S10. Volcano plot from our tissue-specific Mendelian randomization analysis for the very low-density lipoprotein-associated region (rs80026582). Outcome data from [39]. (PDF 1036 kb) [file 13073_2019_613_MOESM1_ESM.pdf]

$$\beta_{Wald\ ratio} = \frac{\hat{\beta}_{Y|Z}}{\hat{\beta}_{X|Z}}$$

$$se(\beta_{Wald\ ratio}) = \sqrt{\frac{se(\hat{\beta}_{Y|Z})^2}{\hat{\beta}_{X|Z}^{\ 2}} + \frac{\hat{\beta}_{Y|Z}^{\ 2} se(\hat{\beta}_{X|Z})^2}{\hat{\beta}_{X|Z}^{\ 4}} - \frac{2\hat{\beta}_{Y|Z} cov(\hat{\beta}_{X|Z}, \hat{\beta}_{Y|Z})}{\hat{\beta}_{X|Z}^{\ 3}}}$$

Figure S1. MR effect estimates are based on the Wald ratio test, where $\hat{\beta}\ Y|Z$ is the coefficient of the genetic variant in the regression of the exposure (e.g. gene expression) and $\hat{\beta}\ X|Z$ is the coefficient of the genetic variant in the regression of the outcome (e.g. cardiovascular trait).

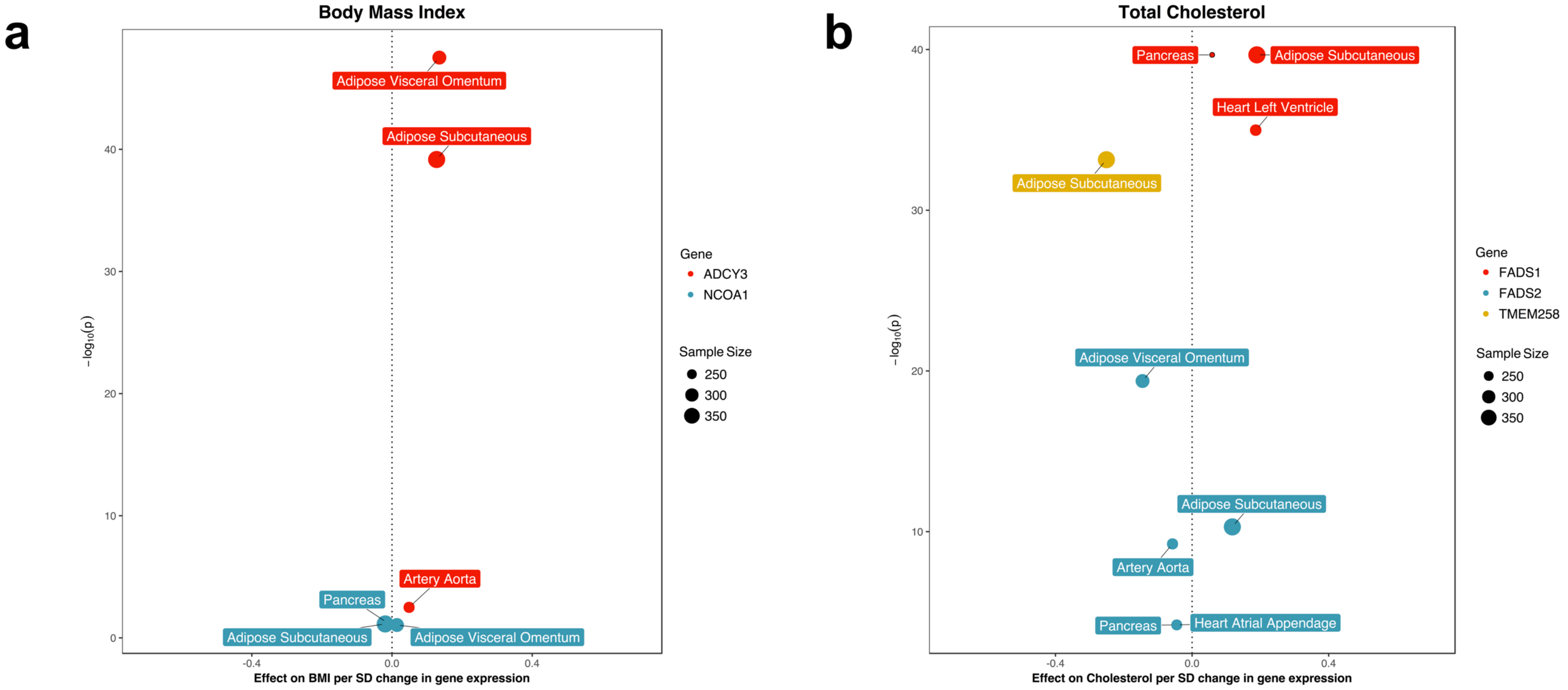

Figure S2. Volcano plots illustrating tissue-specific MR results. Effect sizes and p-values obtained from the MR wald-ratio.
(a) Tissue-specific MR results for the observed effect on BMI. *ADCY3* gene expression provided strong evidence that it influenced BMI in comparison to the *NCOA1* gene.
(b) Tissue-specific MR results for the observed effect on total cholesterol. All 3 genes provided strong evidence of association with total cholesterol at this region across various cardiovascular-specific tissue types.

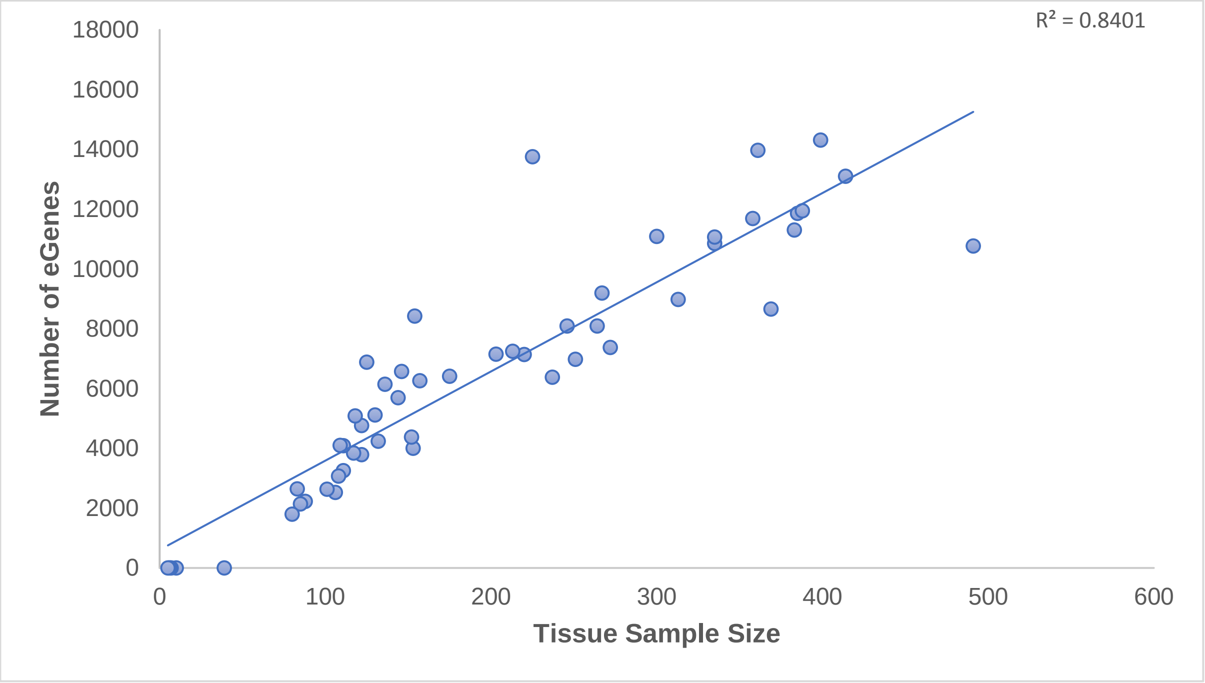

Figure S3. Scatter plot illustrating how eGene discovery increases as sample size increases (R^2 = 0.84). Figure adapted from the Genotype Tissue Expression Project [15].

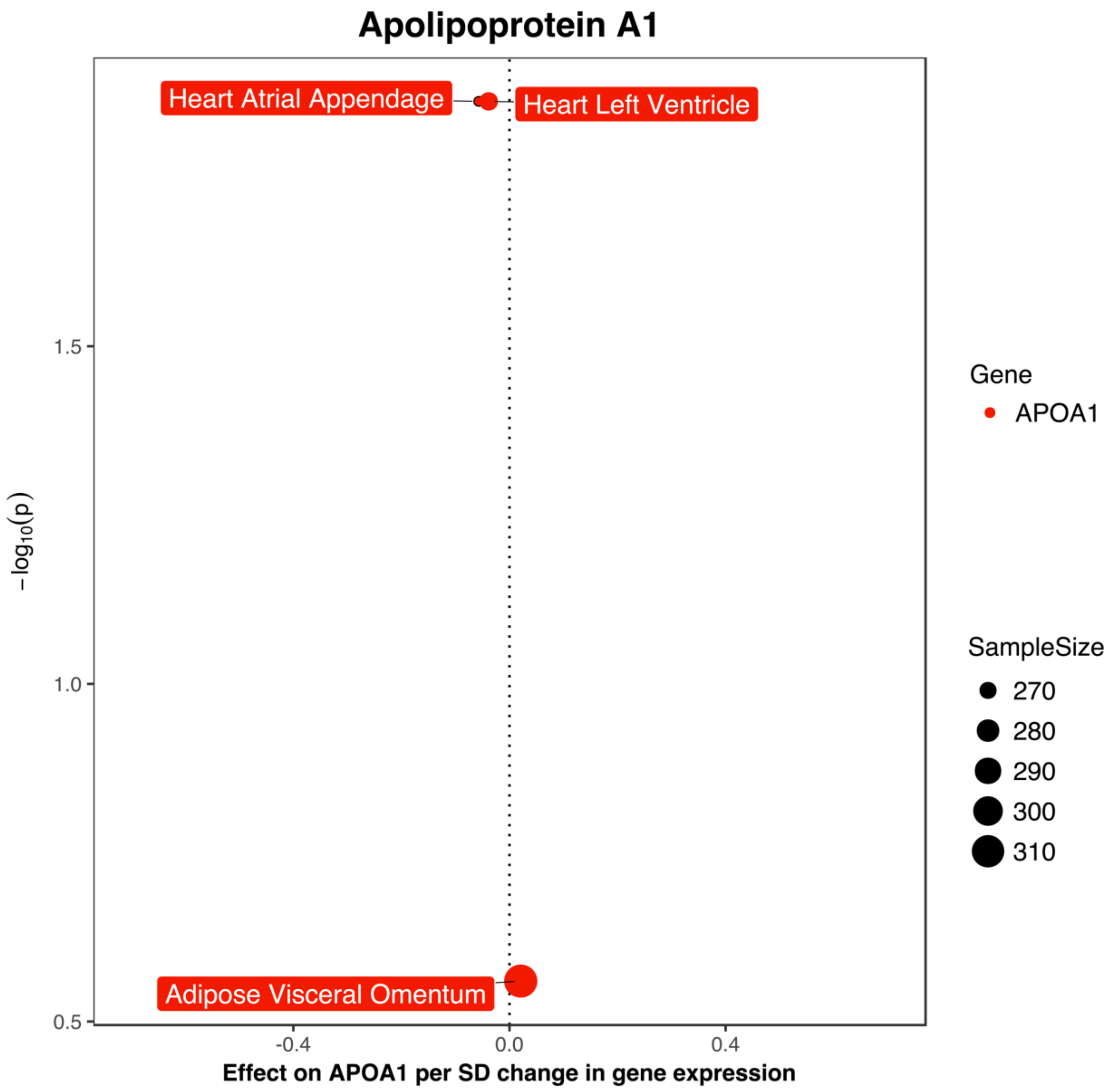

Figure S4. Volcano plot from our tissue-specific Mendelian randomization analysis for the Apolipoprotein A1 associated region (rs2727784). Outcome data from Kettunen et al (2016).

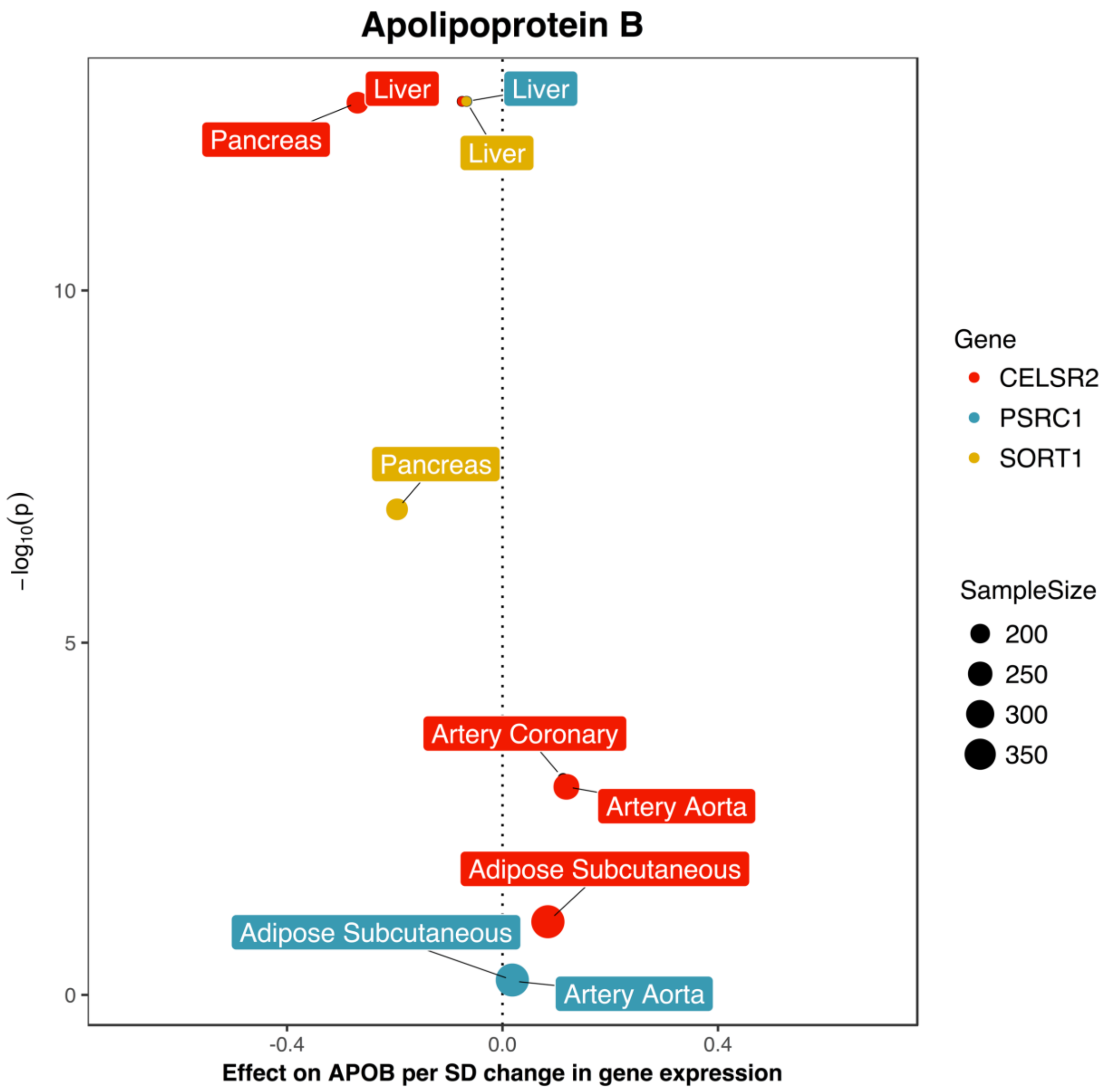

Figure S5. Volcano plot from our tissue-specific Mendelian randomization analysis for the Apolipoprotein B associated region (rs646776). Outcome data from Kettunen et al (2016).

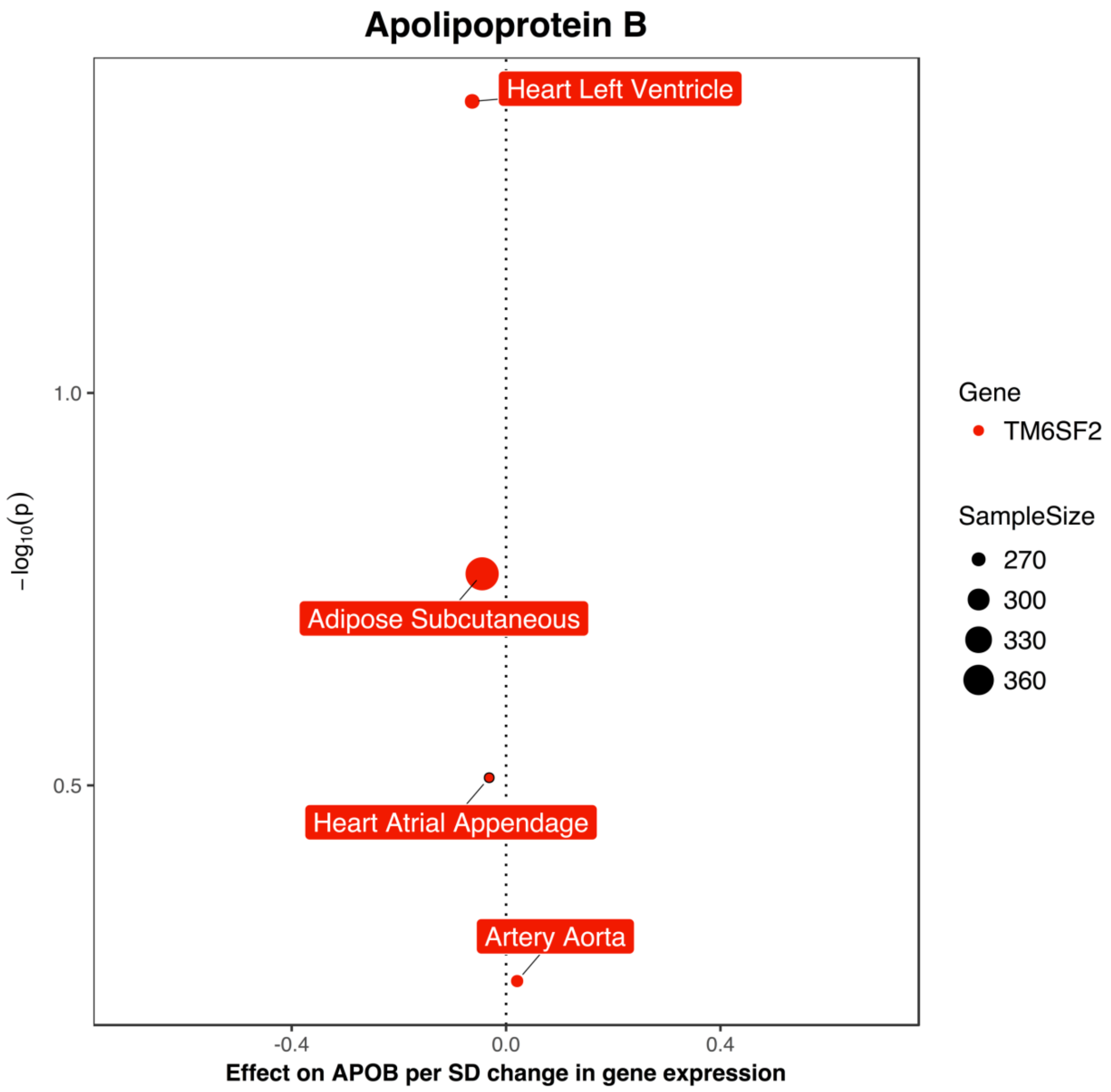

Figure S6. Volcano plot from our tissue-specific Mendelian randomization analysis for the Apolipoprotein B associated region (rs10419998). Outcome data from Kettunen et al (2016).

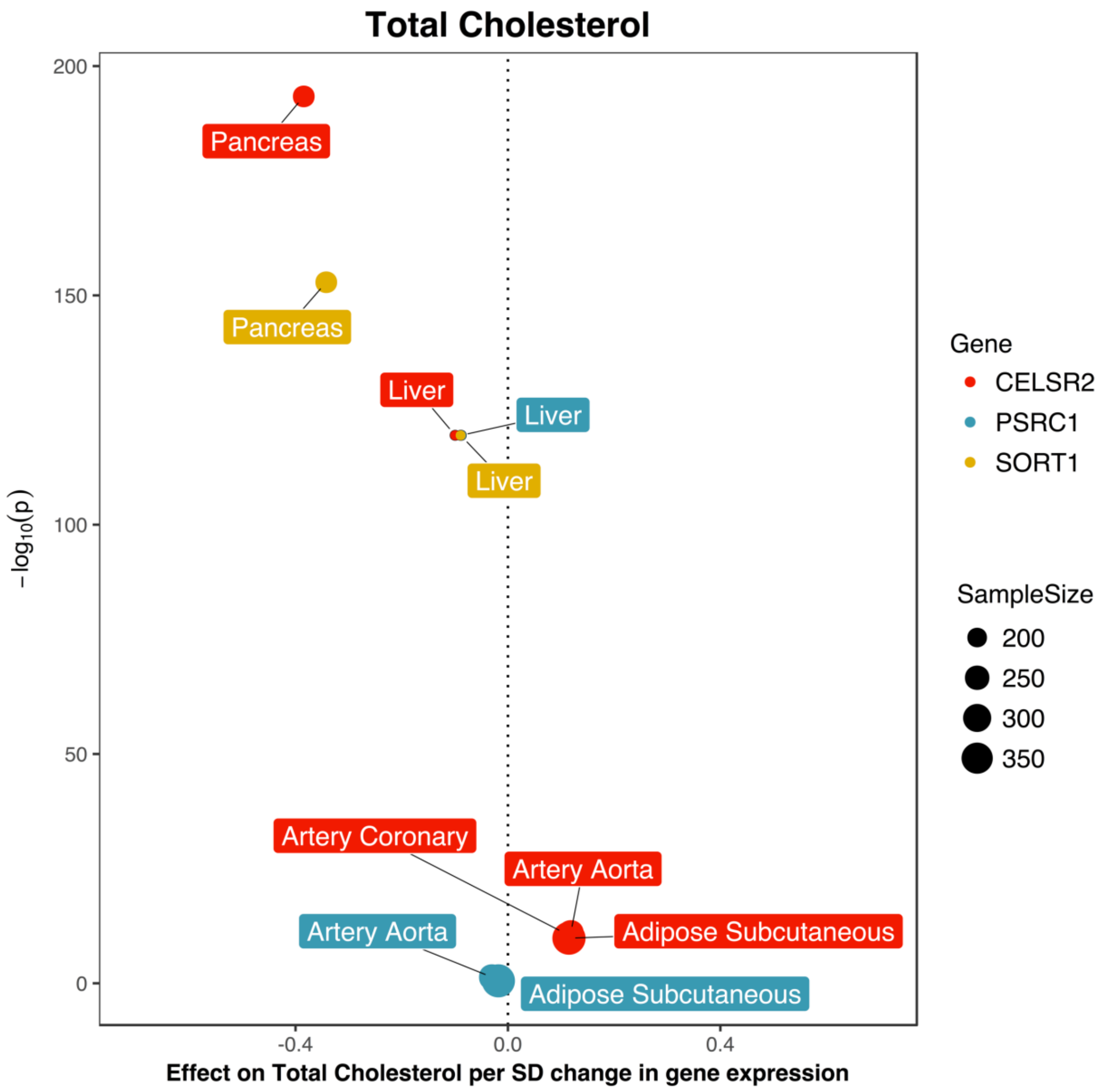

Figure S7. Volcano plot from our tissue-specific Mendelian randomization analysis for the cholesterol associated region (rs646776). Outcome data from Willer CJ et al (2016).

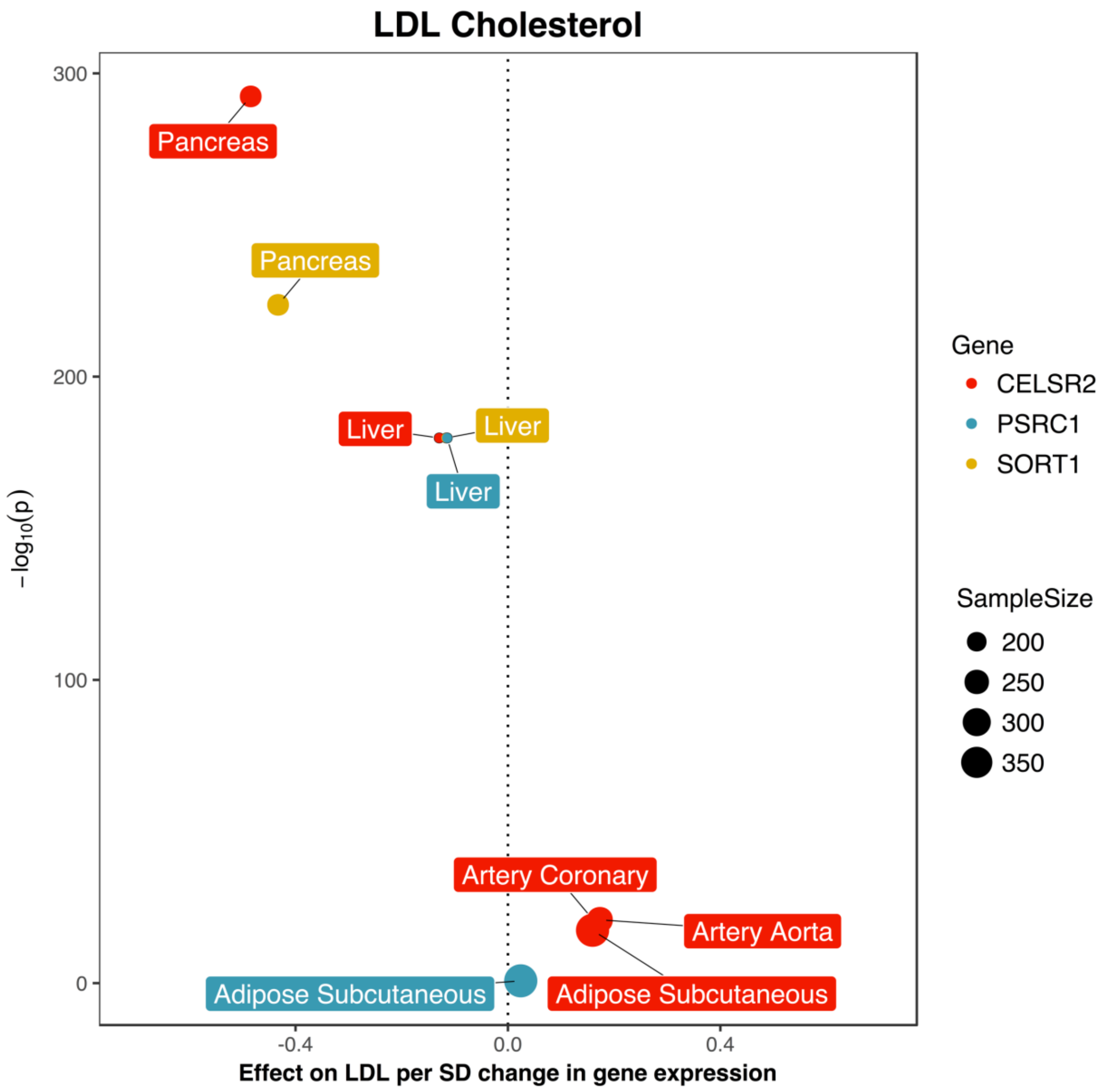

Figure S8. Volcano plot from our tissue-specific Mendelian randomization analysis for the low density lipoprotein associated region (rs646776). Outcome data from Willer CJ et al (2016).

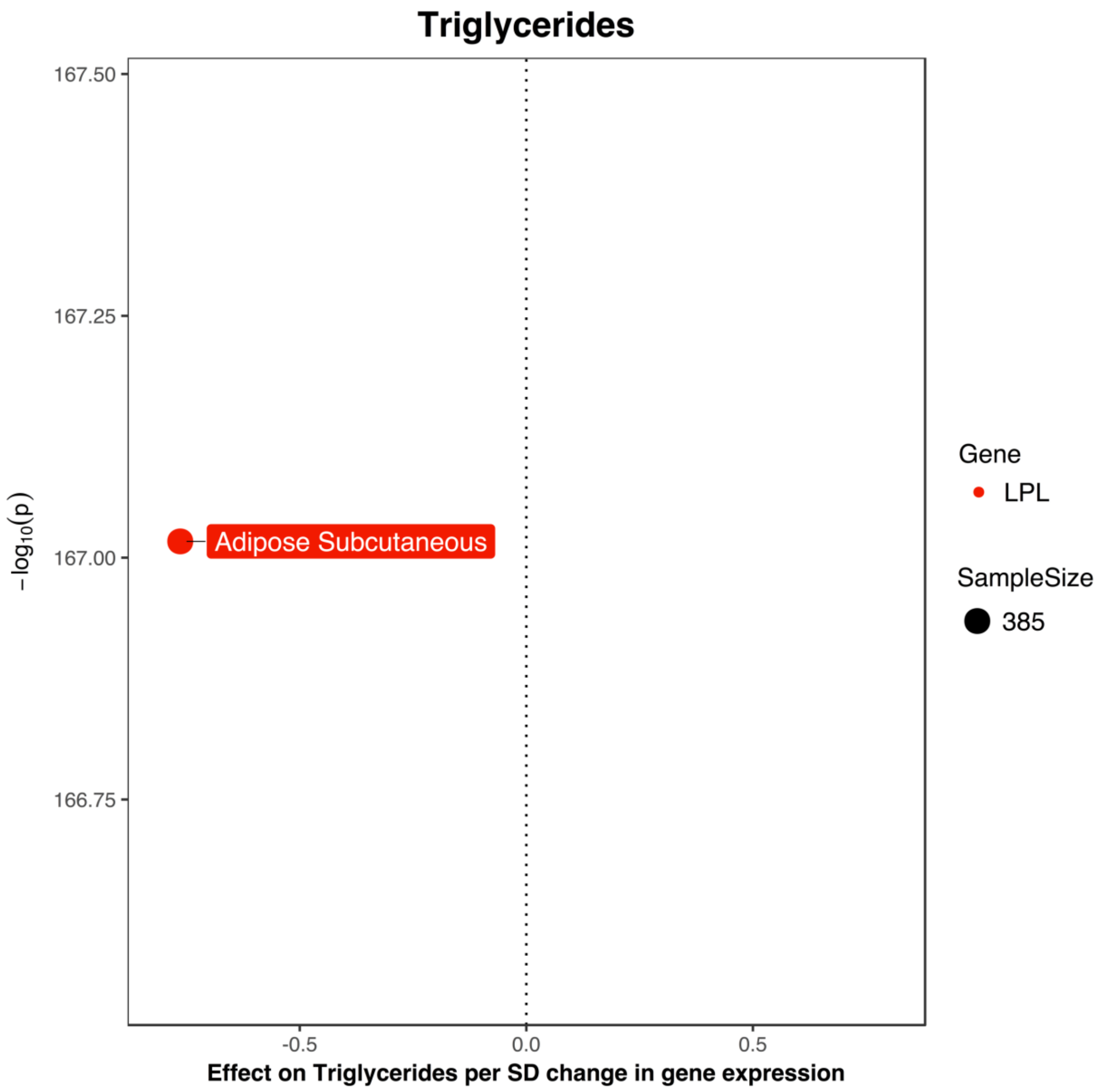

Figure S9. Volcano plot from our tissue-specific Mendelian randomization analysis for the triglyceride associated region (rs80026582). Outcome data from Willer CJ et al (2016).

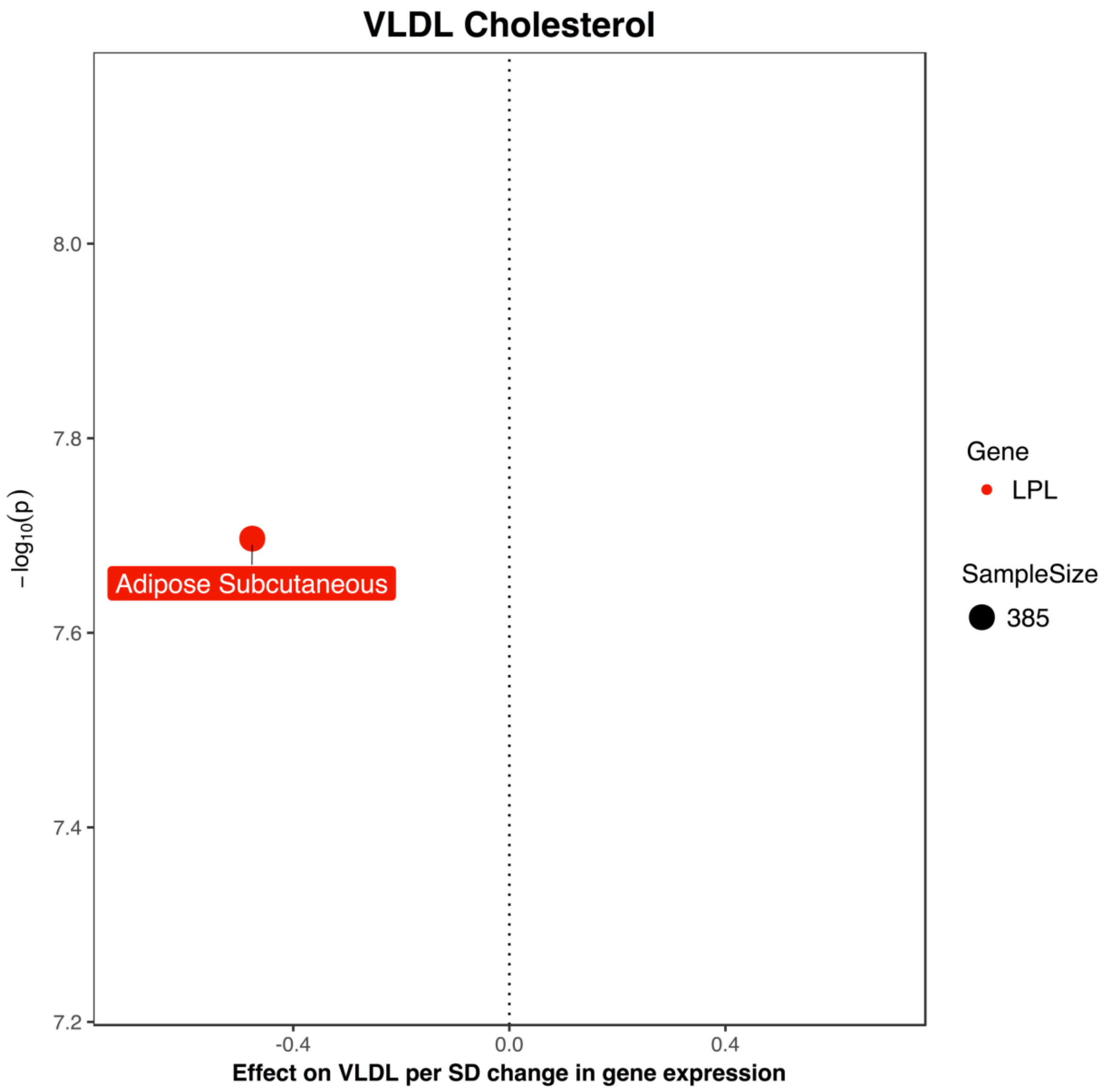

Figure S10. Volcano plot from our tissue-specific Mendelian randomization analysis for the very low density lipoprotein associated region (rs80026582). Outcome data from Ketunnen et al (2016).
